# Transient colonization by *Priestia megaterium* B1L5 alters the structure of the rhizosphere microbiome towards potential plant beneficial bacterial groups in apple plantlets

**DOI:** 10.1186/s40793-025-00762-x

**Published:** 2025-08-13

**Authors:** Fatma M. Mahmoud, Holger Edelmann, Yang Si, Lea Endrejat, Karin Pritsch, Caroline Gutjahr, Armin Ehrenreich, Traud Winkelmann, Jana Barbro Winkler, Jörg‑Peter Schnitzler, Michael Schloter

**Affiliations:** 1 Research Unit for Comparative Microbiome Analysis, Helmholtz Munich, German Research Center for Environmental Health, Neuherberg, Germany; 2 Chair of Microbiology, TUM School of Life Sciences, Technical University of Munich, Freising, Germany; 3 Plant Genetics, TUM School of Life Sciences, Technical University of Munich, Freising, Germany; 4 Max-Planck-Institute of Molecular Plant Physiology, Potsdam-Golm, Germany; 5 Research Unit for Environmental Simulations, Helmholtz Munich, German Research Center for Environmental Health, Neuherberg, Germany; 6 Institute of Plant Genetics, Leibniz University Hannover, Hanover, Germany; 7 Chair of Environmental Microbiology, TUM School of Life Sciences, Technical University of Munich, Freising, Germany; 8 Botany and Microbiology Department, Faculty of Science, Suez Canal University, Ismailia, Egypt

**Keywords:** Apple replant disease (ARD), Plant growth-promoting bacteria (PGPB), GFP-labelled mutant, Root colonization, Rhizosphere microbial community, Metabarcoding

## Abstract

**Background:**

Plant growth-promoting bacteria (PGPB) can beneficially modulate rhizosphere microbial communities, potentially improving plant health and reducing disease incidence. Limited research exists on the influence of PGPB inoculation on the rhizosphere microbial communities of apple plants, particularly in soils affected by apple replant disease (ARD). Here, we evaluated the capacity of GFP-labelled *Priestia megaterium* B1 (designated as *P. megaterium* B1L5) to colonize the roots of apple plantlets grown in two soils: ARD-affected soil and ARD-unaffected grass soil. We investigated its influence on plant growth in ARD-affected soil and its potential to mitigate ARD-related symptoms. We also assessed how its inoculation modulates the rhizosphere microbial communities, with emphasis on changes that may support plant health, particularly in ARD-affected soils.

**Results:**

*P. megaterium* B1L5 successfully colonized apple roots in both soils 6 days post-inoculation (dpi), but was not detectable at 33 dpi. In ARD-affected soil, plants inoculated with vegetative cells or spores displayed a lower proportion of blackened root tips compared to uninoculated controls. Beta diversity and PERMANOVA analyses demonstrated a significant influence of inoculation on the bacterial communities in both soils at 6 and 33 dpi (*p* = 0.001). Furthermore, inoculation enriched the rhizosphere of apple plantlets with potential plant-beneficial bacteria, such as *Luteimonas*, *Lysobacter*, *Pseudomonas*, *Sphingomonas*, *Sphingobacterium*, *Rhodanobacter*, *Pedobacter* and *Flavobacterium*. In contrast, fungal communities remained largely unaffected by inoculation. Most bacterial and fungal shifts observed in the rhizosphere of inoculated plantlets at 33 dpi did not exhibit similar patterns in uninoculated controls over time, indicating that these shifts were largely driven by the inoculum rather than by plant development or natural microbial succession.

**Conclusions:**

Our results highlight the capacity of *P. megaterium* B1L5’s to transiently colonize apple plant roots across different soil environments. The observed tendency toward reduced root tip blackening in inoculated plants grown in ARD-affected plants reflects its potential for alleviating stress associated with ARD. Additionally, inoculation with *P. megaterium* B1L5 promoted beneficial shifts in the rhizosphere microbiome by enriching bacterial taxa commonly linked to plant health. These findings indicate that *P. megaterium* B1L5 presents a candidate for ARD mitigation, however its long-term efficacy and practical application should be further evaluated.

**Supplementary Information:**

The online version contains supplementary material available at 10.1186/s40793-025-00762-x.

## Introduction

Plant growth-promoting bacteria (PGPB) have been the focus of extensive research as sustainable, eco-friendly alternatives to chemical fertilizers and pesticides. PGPB enhance plant growth and development through a range of direct and indirect mechanisms, which include the production of phytohormones, improving nutrient acquisition, enhancing plant resistance to stress and suppressing plant pathogens [1]. The effectiveness of PGPB relies heavily on their ability to successfully colonize and establish in the root-associated environment, as well as their interaction with the indigenous rhizosphere microbial communities [2]. Resource competition and the diversity of indigenous microbial communities also influence the survival and persistence of the inoculum [3–5]. Additionally, the survival of inoculated PGPB in the root-associated environment depends on their ability to utilize root exudates [6, 7]. While the inoculated strain must meet its nutritional needs from exuded compounds, it must also withstand compounds that could be inhibitory like phenolic compounds (e.g. phytoalexins [8]). Importantly, root exudation patterns are strongly influenced by the surrounding soil environment [9] as well as abiotic and biotic stresses [10]. In return, inoculation with PGPB can modify native communities of the rhizosphere not only through direct interaction with resident microbes but also indirectly by altering root exudation patterns [2]. These complex interactions between inoculum, native microflora and plant responses make it challenging to predict the success of PGPB inoculations and the subsequent consequences for plant health.

Apple replant disease (ARD) is a phenomenon that affects apple production worldwide, resulting in damaged root systems, reduced shoot growth and diminished fruit quality and quantity. ARD typically occurs when apple trees are replanted at the same sites successively [11, 12]. Plants facing ARD show an accumulation of phytoalexins in root tissues, which they also exude into the root vicinity [13]. The improvement in plant growth following fungicide application, soil pasteurization or fumigation demonstrated that biological agents are involved in disease development [11, 14]. Somera and Mazzola (2022) described ARD as a disease that develops due to plant-induced alterations in the soil microbiome, which facilitate the proliferation of a synergistic complex of soil-borne pathogens [15]. Earlier studies comparing root-associated microbial communities of apple plantlets grown in ARD soil with those in grass soil, with no history of apple cultivation, revealed changes in microbial communities, a phenomenon referred to as dysbiosis [16, 17]. The pathogenic complex underlying ARD generally comprises fungi, oomycetes, and nematodes [15]. However, the abundance and contribution of these pathogens to disease development and severity vary across geographic locations, orchards and between years [15]. Among the fungal genera, *Rhizoctonia* and *Cylindrocarpon* were linked to ARD [11, 18]. The role of *Fusarium* in ARD is still debated, though it was frequently recovered from ARD soil [11, 19]. While Mazzola (1998) showed that inoculation with *Fusarium* had a weak or no negative influence on plant growth [11], other studies demonstrated a positive correlation between the abundance of *Fusarium* and ARD severity [20, 21]. Analyses of fungal communities of apple plantlets cultivated in replanted soil revealed the enrichment of *Ilyonectria* [16, 22], *Thelonectria* [22], *Nectria* sp. [16], *Cylindrocarpon* and *Fusarium* [21]. Genera of oomycetes including *Pythium* and *Phytophthora*, as well as the nematode *Pratylenchus* were reported to contribute to ARD development [15]. Additionally, a metagenomic analysis of microbial communities in ARD soil highlighted its competitive and stressful nature, underscored by the increased abundance of genes involved in antibiotic synthesis and stress sensing [23].

Despite the effectiveness of chemical fumigants [24], their toxicity and detrimental effects on soil ecology highlight the need for sustainable eco-friendly alternatives to mitigate ARD. Crop rotation [25], biofumigation [26], anaerobic soil disinfection [27] and soil amendments [28] have been investigated as sustainable alternatives to mitigate ARD. Additionally, there is a growing interest in using PGPB to alleviate ARD symptoms [29–34], though only few studies assessed the influence of the inoculation on soil and root-associated microbial communities [22, 32, 33]. Microbial inoculation can positively modulate the indigenous microbiome by reversing dysbiosis (countering changes driven by pathogens), promoting beneficial native microbes or inhibiting potential pathogens [35]. Since plant-induced microbial imbalances are key contributors to ARD, modulation of microbial communities could play a role in mitigating ARD. However, further research is still needed to better understand how inoculants influence the rhizosphere and root-associated microbiome of plants experiencing ARD and how this impacts plant health.

*Priestia megaterium* (formerly known as *Bacillus megaterium* [36]), a Gram-positive spore-forming bacterium [37], has been reported to enhance plant growth and yield in various economically important crop plants, including rice [38], wheat [39], maize [40] and potato [41]. Additionally, it was able to increase plant tolerance and enhance plant growth under abiotic [42, 43] or biotic stresses [44, 45]. *P. megaterium* strain B1 was isolated from the roots of healthy apple plants grown in gamma-irradiated grass soil without ARD background [46]. Genomic and physiological analysis of strain B1 revealed its potential to colonize plant roots and promote plant growth [47]. Additionally, its genome revealed the surfactin biosynthetic gene cluster, suggesting an antimicrobial potential. Taken together, these features highlight the potential of *P. megaterium* B1 to colonize apple roots and function as a PGPB that supports plant health. However, since it was originally isolated from the roots of apple plants grown in a reduced-microbial soil system unaffected by ARD, its capacity to persist and function under ARD conditions requires evaluation.

Here, we conducted a plant experiment under controlled conditions in the sun simulator facility of Helmholtz Munich (https://www.helmholtz-munich.de/en/eus/research-groups/research-group/our-facilities/sun-simulators) [48, 49]. We constructed a GFP-labelled mutant of *P. megaterium* B1, designated as strain *P. megaterium* B1L5 and inoculated the roots of apple plantlets with either its vegetative cells or spores. We then grew them in in two soils: one with a history of apple monoculture and inducing ARD (ARD-affected) and one with no history of apple cultivation (ARD-unaffected grass soil). Our main goals were to assess the colonization and establishment of *P. megaterium* B1L5 in the apple root-associated environment (6 and 33 dpi) and to investigate its potential role in mitigation of ARD symptoms. We also investigated the influence of inoculation on the rhizosphere bacterial and fungal communities, to understand how it may modulate rhizosphere microbial communities, with a particular focus on how these microbial changes could potentially support plant health, especially in the context of ARD. Based on the physiological and genomic traits of *P. megaterium* B1, we hypothesized that this strain will effectively colonize apple roots (H1). We also anticipated that it will enhance plant growth and reduce ARD symptoms (H2). Additionally, we expected that it will alter both bacterial and fungal communities in the rhizosphere (H3).

## Methods

### Construction of GFP-labelled strain *P. megaterium* B1L5

All bacterial strains were grown on LB with 10 g/L trypton, 5 g/L yeast extract and 5 g/L NaCl. Higher salt concentrations inhibited the growth of the used *Bacillaceae* strains. Used strains and plasmids are listed in Tables 1 and 2.

**Table 1:** Used bacterial strains

Name	Description	Origin
*Bacillus subtilis*	Laboratory strain 168	DSMZ (DSM 402)
*Priestia megaterium* B1	Laboratory strain (GeneBank CP141294.1)	Mahmoud et al. (2023) [46]
*Priestia megaterium* B1L5	xylR-P_groES_-sfGFP-His_6_	this work
*E. coli* CA434	*F- mcrB mrr hsdS20(rB- mB-) recA13 leuB6 ara-14 proA2 lacY1 galK2 xyl-5 mtl-1 rpsL20 (SmR) glnV44 λ-)*	University of Nottingham (online store)
*E. coli* DH10 β	F^–^*mcr*A Δ(*mrr*-*hsd*RMS-*mcr*BC) φ80*lac*ZΔM15 Δ*lac*X74 *rec*A1 *end*A1 *ara*D139 Δ(*ara-leu*)7697 *gal*U *gal*K λ^–^*rps*L(Str^R^) *nup*G	New England Biolabs GmbH (NEB)

**Table 2:** Used plasmids

Name	Description	Origin
pKVM4	*oriT*, *traJ*, *ampR*, *ermR*, oriR(pE194ts), P*clpB*-*codBA*, oriR(pBR322)	Kostner et al. (2017) [50]
pBACOV-sfGFP-His6	*oriT*, *traJ*, *kanR*, *ampR*, ori ColE1, ori(pUB110) P_aprE_-*sfGFP*-His_6_	Heinze et al. (2018) [51]
pPRIM1	pKVM4, NQ126_016855::P_GroES_-*sfGFP*-His_6_	This work

A markerless insertion of GFP on the chromosome of *P. megaterium* B1 strain was constructed by allelic exchange based on the conjugative plasmid pKVM4 using 5-fluorocytosin (5-FC) counter selection [50, 51]. Schematic representation of the complete workflow used in this study for construction of strain *P. megaterium* B1L5 from *P. megaterium* B1 is given in (Figure S1). The insertion locus after the terminator of *xylR* should not affect other gene functions. Expression of the *sfGFP-his*_*6*_ gene was achieved by a strong *groES*-promotor from *B. subtilis* 168 (DSM402), combined with the *gfp* gene and terminator of pBACOV-sfGFP [52]. The expression cassette flanked by two regions of 1 kb and 1.6 kb length, homologous to the chromosomal sequence of *P. megaterium* B1 was inserted into the plasmid pKVM4, resulting in the plasmid pPRIM1 (Figure S2). The flank size difference resulted from a sequencing error in the old genome sequence. Used genome and plasmid sequence can be found at NCBI with GenBank number CP141294.1, AL009126.3 and MG599121.1. Custom DNA primers (ordered by Sigma-Aldrich Chemie GmbH, Taufkirchen, Germany) were used to generate five linear DNA fragments with homologous ends (Tables S1 and S2) using Q5 polymerase (New England Biolabs GmbH (NEB), Frankfurt am Main, Germany). The backbone fragment 4 was *Dpn*I (NEB, Frankfurt am Main, Germany)-digested to reduce the amount of false positive colonies. All fragments were gel extracted and then fragments 1–5 were ligated using Gibson Assembly Mix (NEB, Frankfurt am Main, Germany) according to the manufacturer’s instructions. After heat shock transformation of 2 µL reaction mix into *E. coli* DH10β generated plasmids were verified by restriction digest and Sanger sequencing (GENEWIZ, Leipzig, Germany).

Triparental conjugation was used to transform *P. megaterium* B1 with pPRIM1. Overnight cultures of *E. coli* CA434 (helper), *E. coli* DH10β with a pKVM4 derivative (donor) and acceptor were grown in LB medium supplemented with 10 mg/L tetracycline, 100 mg/L carbenicillin, and w/o antibiotic. On the next day fresh cultures in respective medium were harvested when optical density at 600 nm (OD_600_) reached 0.8. Two milliliters of each culture were centrifuged at 2500 x g at 4 °C, the pellets were washed once with cold LB and resuspended in LB. After combination of strains and additional centrifugation, cells were resuspended in 150 µL of fresh LB and dropped onto a LB plate without antibiotic. After incubation for at least 20 h at 37 °C, the cell material was resuspended in 600 µL LB medium, pasteurized at 70 °C for 15 min and spread on four LB plates with 5 mg/mL erythromycin. The plates were then incubated at 30 °C for 1–3 days until colonies appeared.

Chromosomal integration of the plasmid was forced by incubation of transconjugants on LB plates at 42 °C to inactivate the temperature sensitive origin of replication of the plasmid. Counter selection due to the vector-encoded *codBA* genes was then done by streaking on half-strength nutrient broth (NB) agar plates (final 4 g/L, BD Difco™) with 60 mg/ml 5-FC. Positive clones were identified by PCR using genome-specific primers, streaked again onto counter-selection plates, and screened for 5-FC^R^ and Erm^S^ phenotypes. The correct chromosomal sequence was then verified by Sanger sequencing.

### Used soils

Soils were collected at a site in Heidgraben, Germany (x-coordinate 53.699199; y-coordinate 9.683171; WGS 84, Schleswig-Holstein, northern Germany). Soil texture, physical and chemical characteristics were described by Mahnkopp et al. (2018) [53]. The soil was classified as sand (medium sand), with a pH of 5.3, soil organic carbon (SOC) content of 25.4 g kg^− 1^, and total nitrogen of 1.45 g kg^− 1^. The site, established in 2009, comprises ARD plots where apple rootstock ‘Bittenfelder Sämling’ was replanted every two years. Previous studies using soil from these plots confirmed the development of apple replant disease (ARD) [53, 54]; this soil is therefore referred to as ARD-affected. The site also include grass plots with no history of apple cultivation [53] and the soil is denoted as ARD-unaffected grass soil. Topsoil (0–20 cm) was collected in December 2023 as a pooled sample from 3 points in ARD or grass plots, where approximately 35 L were collected for each type of soil. The soil was then stored at 4 °C for one week prior to the start of the experiment. The soil was homogenized by sieving through a 4.5-mm mesh and fertilized with 2 g L⁻¹ Osmocote Exact 3–4 M (16% *N* + 9% P_2_O_5_ + 12% K_2_O + 2% MgO, ICL Deutschland, Nordhorn, Germany) before planting.

### Preparation of bacterial inoculants

To propagate vegetative cells *P. megaterium* B1L5 was cultured in NB medium (Carl Roth, Karlsruhe, Germany) and incubated at 30 °C with shaking at 120 rpm. After 18 h, cells were harvested by centrifugation for 10 min at 3270 x g (Allegra X-12, Beckman Coulter, IN, USA), washed twice by 1x PBS (AppliChem, Darmstadt, Germany) and then resuspended in sterile tap water. Harvesting was done on the planting day to ensure cell viability. The colony-forming units (CFU) of the vegetative cell inoculant were estimated by plate counting and measuring OD_600_.

For spore propagation, *P. megaterium* B1L5 was inoculated in half-strength NB medium (Carl Roth, Karlsruhe, Germany) supplemented with MnSO_4_ (5 mg/L) [37] and incubated at 30 °C for 6 days with shaking at 120 rpm. Cells were harvested by centrifugation for 10 min at 3270 x g (Allerga X-12, Beckman Coulter, IN, USA) and washed twice by PBS (AppliChem, Darmstadt, Germany), then resuspended in sterile tap water. To kill vegetative cells and facilitate sporulation suspension was heated at 65 °C for 1 h [55]. Spore formation was confirmed by phase-contrast microscopy, and spore counts were estimated by plate counting of the heated suspension.

### Plant material

Plants of the ARD-susceptible rootstock genotype M26 were propagated in vitro as described by Mahnkopp et al. (2018) [53]. Briefly, the shoots were propagated every five weeks through axillary shoot formation on Murashige and Skoog (MS) medium [56] containing 3% sucrose, 4.4 µM BAP (6-benzylaminopurine), 0.5 µM IBA (indole-3-butyric acid), and 0.8% Plant Agar (Duchefa, The Netherlands), with the pH adjusted to 5.7. For rooting, single shoots were transferred to a half-strength MS medium comprising 2% sucrose, 4.92 µM IBA, and 0.75% Plant Agar at pH 5.7. After 3 weeks, plants were acclimatized in a commercial peat substrate (Steckmedium, Klasmann-Deilmann GmbH, Geeste, Germany).

### Climate chamber experiment

The experiment was performed under controlled conditions in the sun simulator facility of Helmholtz Munich (https://www.helmholtz-munich.de/en/eus/research-groups/research-group/our-facilities/sun-simulators) [48, 49]. On the planting day, 12 weeks-old plantlets were carefully removed from the peat substrate, ensuring minimal disturbance. Any remaining substrate on the roots was gently washed off using tap water. The roots of the plantlets were then immersed, for 10 min, in 10 mL of either vegetative cells or spore suspensions with a concentration of 10⁷ CFU/mL. Control plantlets were dipped in sterile tap water. Following this, the plantlets were transplanted into pots (10 × 10 × 11 cm) containing 600 g of either ARD soil or grass soil (1 plantlet/pot). Afterwards, the whole volume of the respective suspensions (10 mL) of either vegetative cells, spores or control sterile tap water was added to the soil around the stem. The treatments are named throughout the manuscript as “Control”, “Vegetative cells” and “Spores”, for control plants and plants inoculated with either vegetative cells or spores, respectively. Six replicates were prepared for each soil, treatment, and time point. The pots were then placed in trays and kept in climate chambers under controlled conditions with temperatures of 22 °C during the day and 18 °C at night, a 16-hour light period with a photosynthetic active radiation (PAR) of 150 µmol photons m⁻² s⁻¹ and air humidity levels between 50 and 70%. The experiment was conducted over 33 days. The plantlets were watered every two days with 50–70 mL of tap water, ensuring the soil remained moist without water leakage.

### Sampling

Sampling was performed at 6 and 33 days post-inoculation (dpi) to capture both early and later stages of root colonization by *P. megaterium* B1L5. The 6-day time point was selected based on previous studies [57, 58] as it represents an early colonization window (first week post inoculation) when *P. megaterium* B1L5 was expected to interact with the young root system and establish initial colonization. The 33-day time point was chosen to assess the persistence of the inoculant at a more developed stage of root growth, informed by studies that detected inoculated bacteria up to 30 days and 5 weeks post-inoculation, but with decreased cell counts [57, 58]. Additionally, this time point aligns with the timeframe used in previous studies employing short-term ARD bioassays (28–34 days), which have demonstrated the effects of ARD on shoot growth, root development and root-associated microbial communities [16, 17, 59]. Sampling was done on each replicate of each treatment, with every replicate consisting of one plant per pot. The roots of each plant were carefully removed from the soil with rhizosphere soil still attached and placed on an alcohol-disinfected tray. Rhizosphere soil (closely adhering to the roots) was gently shaken off, thoroughly mixed, and transferred into sterile 1.5 mL tubes and immediately placed on dry ice. Roots were then washed by gentle dipping in sterile tap water. At least 3–5 representative roots per plant/per treatment were cut using sterile scalpels and collected on autoclaved moist filter paper in a sterile Petri dish for microscopic examination of colonization by *P. megaterium* B1L5. The remaining roots were collected in sterile 1.5 mL tubes and immediately placed on dry ice. Rhizosphere soil and root samples for DNA extraction were preserved at -80 °C. At the second harvest, 33 dpi, additionally plant growth parameters including shoot length, shoot fresh mass and root fresh mass were recorded. High-quality photos of the root system were taken immediately after sampling and prior to sectioning. As the roots needed to be processed quickly for microscopic examination, these images were used to count blackened and white root tips. Root tips were classified as blackened if they showed visible dark discoloration or necrosis (characteristic for ARD-affected roots [60]), in contrast to healthy root tips, which remained white. Counting was performed for all replicates of each treatment. The percentage of blackened root tips was calculated as: (number of blackened tips / total number of root tips) × 100.

### Microscopic examination of roots

Roots assigned for microscopic evaluation were examined directly on the sampling day at 6 and 33 dpi. Representative sections of the different root zones, including absorptive, transitionary and transportive roots, were selected for examination [61]. Roots were cut into 1 cm pieces, placed on clean sterile slides with a drop of water, and covered with coverslips. At least 3 replicates were examined for each treatment, with 3–5 root pieces examined per replicate. Microscopic examination was performed using a Zeiss LSM880 confocal laser scanning microscope (CLSM) (Zeiss, Oberkochen, Germany) equipped with an argon ion laser and a helium-neon laser. The GFP signal was captured using the FITC channel (excitation at 488 nm), while the Cy3 channel (excitation at 561 nm) was used to visualize the background and root structure. Root structures were assigned to red in the final image reconstruction to enhance contrast with the green signal. A 64x C-Apochromat water immersion objective was used for the examination (Zeiss, Oberkochen, Germany) and images were recorded using the Zen Black software Edition 2.3 SP1 FP1 (version 14.0.12.201, Zeiss).

### Quantification of *gfp* gene copy number in root samples using qPCR

DNA was extracted from roots that were ground in liquid nitrogen, following a protocol originally developed by Lueders et al. (2004) [62] and modified by Stempfhuber et al. (2017) [63]. The *gfp* gene was quantified in root samples using SYBR Green^®^-based qPCR assay. The design of the qPCR system for quantifying the gene copy number of the partial *gfp* gene is described in detail in the supplementary material.

### Amplicon sequencing and library preparation for rhizosphere bacterial and fungal community analysis

DNA was extracted from 0.5 g of rhizosphere soil samples using the NucleoSpin Soil Kit (Macherey-Nagel, Duren, Germany) according to the manufacturer’s protocol. The DNA was then quantified using the Quant-iT PicoGreen dsDNA Assay Kit (Thermo Fisher Scientific, Darmstadt, Germany). For bacterial community analysis, the V4 region of the 16 S rRNA gene was amplified using the primer pair 515 F [64] and 806R [65]. For analysis of the fungal community, the ITS3 mix and ITS4 mix primers [66] were used to amplify the fungal internal transcribed spacer (ITS). PCR reactions and library preparations are explained in detail in the supplementary material. Negative controls of DNA extraction and PCR were included in all steps of library preparation and sequencing.

### Processing of amplicon sequencing data and statistical analysis

Demultiplexed sequences were processed using the Galaxy web platform (www.usegalaxy.org; [67]). Initial trimming of the FASTQ files was performed with Cutadapt (Galaxy Version 4.4 + galaxy0) [68], ensuring a minimum read length of 50 bp, followed by quality assessment using FastQC [69]. Subsequent read processing employed the DADA2 pipeline [70] within Galaxy Version 24.1.2.dev0. Both 16S rRNA gene and ITS sequences underwent trimming, with 20 bp removed from the beginning of both forward and reverse reads. The expected error thresholds were set to 3 for forward reads and 4 for reverse reads. The read lengths were truncated to 240 bp for forward reads (both 16 S and ITS) and 200 bp for reverse reads (both 16 S and ITS). Taxonomic classification of the resulting amplicon sequence variants (ASVs) was performed using the SILVA database (SILVA v138.1; [71]) for bacterial 16 S rRNA gene sequences and the UNITE database (UNITE release s16.10.2022; [72]) for fungal ITS sequences. ASVs associated with mitochondrial and chloroplast sequences, as well as those detected in negative controls or present as singletons (ASVs represented by a single read) were removed from the final dataset.

Statistical and microbiome data analyses were conducted using R (v4.3.1; [73]). A phyloseq object was created using ‘phyloseq’ package (v1.46.0 [74]), for subsequent analysis. To normalize the data, scaling with ranked subsampling [75] was employed, using the ‘SRS’ R package (v0.2.3 [76]),. All subsequent analyses were performed using the normalized phylosq object. Alpha diversity metrics, including observed richness, Pielou’s evenness, and the Shannon diversity index, were calculated using the ‘microbiome’ package (v 1.24.0; [77]). Beta diversity was evaluated by constructing a Bray-Curtis distance matrix, with principal coordinate analysis (PCoA) applied for ordination, using ‘phyloseq’ package (v1.46.0 [74]),. The significance of differences in community composition was assessed using PERMANOVA (*p* < 0.05) via the ‘adonis2’ function from the ‘vegan’ package (v2.6-4; [78]). Heatmaps featuring the top 20 classes and genera were generated using the ‘ampvis2’ package (v2.8.7; [79]). Differential abundance was analyzed using the ‘DESeq2’ package (v1.42.0 [80]),. For each soil (grass and ARD), the abundance of bacterial and fungal taxa in inoculated samples (vegetative or spore treatments) was compared to the uninoculated control at each time point (6 dpi and 33 dpi). Additionally, to evaluate natural microbial shifts over time in the absence of the inoculum, the control samples from each soil were compared at 33 dpi vs 6 dpi. Taxa with *p* value < 0.05 were considered as significantly different in abundance.

Data were checked for normal distribution and homogeneity of variance by the Shapiro-Wilks and Bartlett’s tests, respectively. Data that passed these tests were analyzed using ANOVA followed by Tukey HSD test; else the Kruskal-Wallis test was used, followed by Wilcoxon test using ‘rstatix’ package (v0.7.2; [81]), with adjustments for multiple comparisons made using the Benjamini–Hochberg method.

## Results

### Colonization of apple roots by *P. megaterium* B1L5 and plant growth response

Six days post-inoculation, CLSM images showed that *P. megaterium* B1L5 successfully colonized apple roots grown in both grass and ARD soil, regardless of whether they were inoculated as vegetative cells or spores (Figures S3A and S4A). However, at 33 dpi, CLSM images revealed no colonization of roots for both inoculation forms in either grass or ARD soils (Figures S3B and S4B). The *gfp* gene copy numbers were quantified in roots at 6 and 33 dpi. In grass soil, the number of *gfp* gene copies per gram of fresh root was 1.41E + 08 in roots inoculated with vegetative cells and 3.32E + 06 in those inoculated with spore inoculation (Figure S3C). In ARD soil, the number of *gfp* gene copies per gram of fresh root was 8.51E + 07 and 3.22E + 06 when inoculated with vegetative cells and spores, respectively (Figure S4C). At 33 dpi, the qPCR signal was undetectable in the majority of replicates in both ARD and grass soil (Table S3).

Plants grown in untreated ARD-affected soil exhibited a significantly higher percentage of blackened root tips compared to those grown in ARD-unaffected grass soil (Table S4), even though no significant differences in plant growth parameters were observed (Table S5). Notably, inoculation with either vegetative cells or spores of *P. megaterium* B1L5 in ARD-affected soil was associated with a reduced (non-significant) proportion of black root tips (Table S4). There was no significant influence of inoculation on plant growth parameters (Table S5).

### Effect of inoculation with *P. megaterium* B1L5 on bacterial communities of the rhizosphere

#### Diversity of rhizosphere bacterial communities

**Fig. 1:**
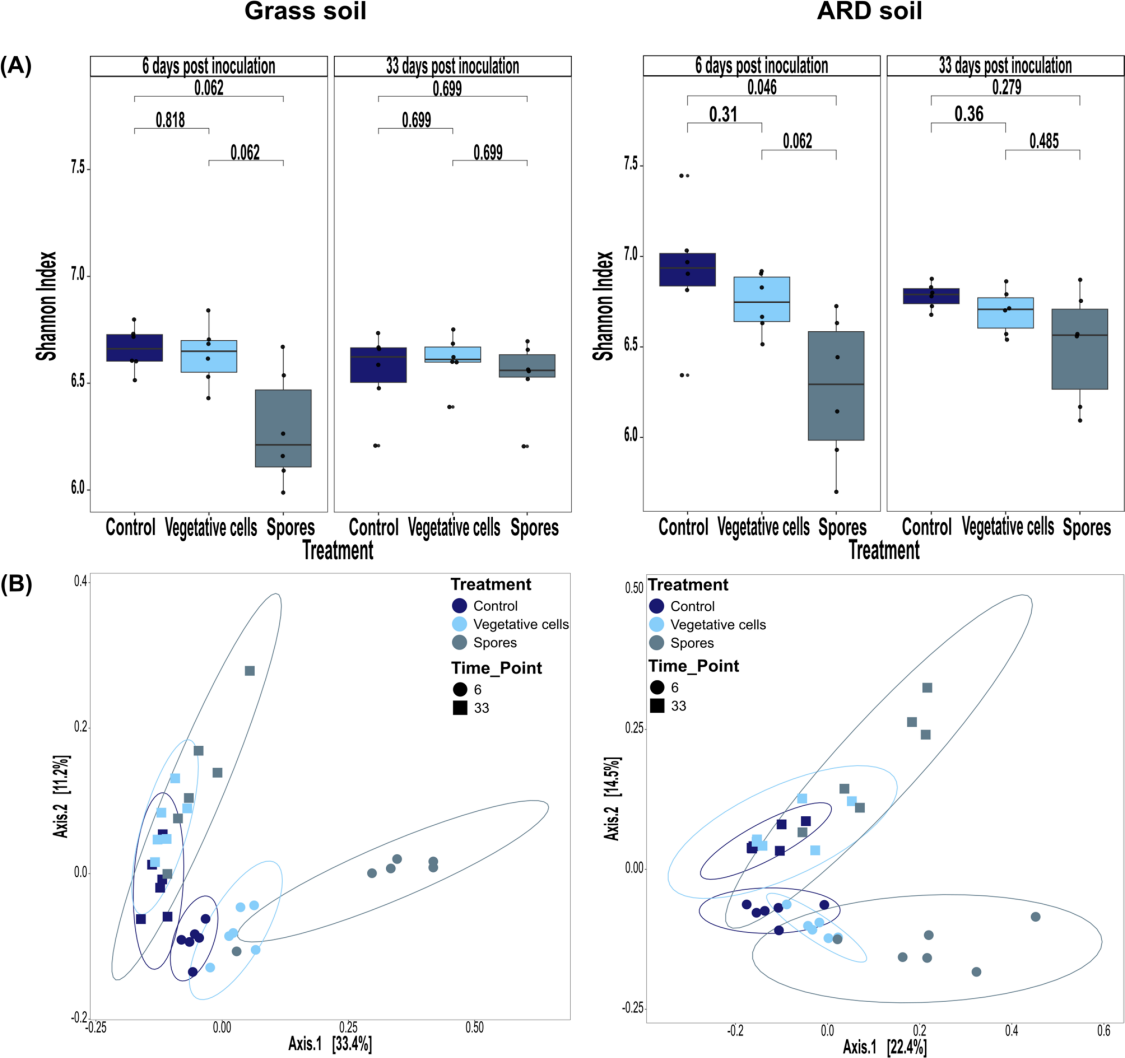
Diversity of rhizosphere bacterial communities of apple plantlets grown in grass or ARD soil. Plantlets were inoculated with either vegetative cells or spores of *P. megaterium* B1L5 or remained uninoculated as control and sampled at 6 and 33 dpi. (**A**) Alpha diversity, represented by Shannon index; significance (*p* < 0.05) was determined using the Wilcoxon test, and adjusted for multiple comparisons using the Benjamini-Hochberg (BH) method. (**B**) Beta diversity of rhizosphere bacterial communities demonstrated by principal coordinates analysis (PCoA) based on Bray–Curtis distance

A total number of 2,676,096 high-quality filtered, normalized reads were assigned to 14,056 bacterial ASVs. In grass soil, alpha diversity of bacterial communities, represented by Shannon, Observed ASVs and Pielou indices, did not differ significantly among the treatments at both time points (Fig. 1A, Figures S5A & S5B).

In ARD soil, Shannon index was significantly lower after spore inoculation compared to the controls at 6 dpi (Fig. 1A). Similarly, Pielou index indicated a significant decrease in evenness in “Spores” treatment compared to “Control” and “Vegetative cells” treatments (Figure S5B). Richness, demonstrated by Observed index, did not differ significantly in treated plants compared to controls (Figure S5A). At 33 dpi, no significant effects of treatments were observed on bacterial alpha diversity indices (Figs. 1A, S5A & S5B).

The composition of bacterial communities was significantly influenced by inoculation with vegetative cells or spores, regardless of the soil type (Fig. 1B). Principal coordinate analysis (PCoA) revealed bacterial communities from different treatments to distinctly cluster with minimal overlapping, particularly at 6 dpi. Additionally, rhizosphere bacterial communities at 6 dpi were clearly separated from those sampled at 33 dpi. PERMANOVA analysis revealed that treatment had a significant effect on the composition of bacterial communities (“grass soil: R2 = 0.19, F = 5.28, *p* value = 0.001”, “ARD soil: R2 = 0.19, F = 4.53, *p* value = 0.001”) (Table S6). Moreover, the composition of bacterial communities of all treatments differed significantly between grass and ARD soils (*p* value = 0.001) (Figure S6).

#### Taxonomic composition

**Fig. 2:**
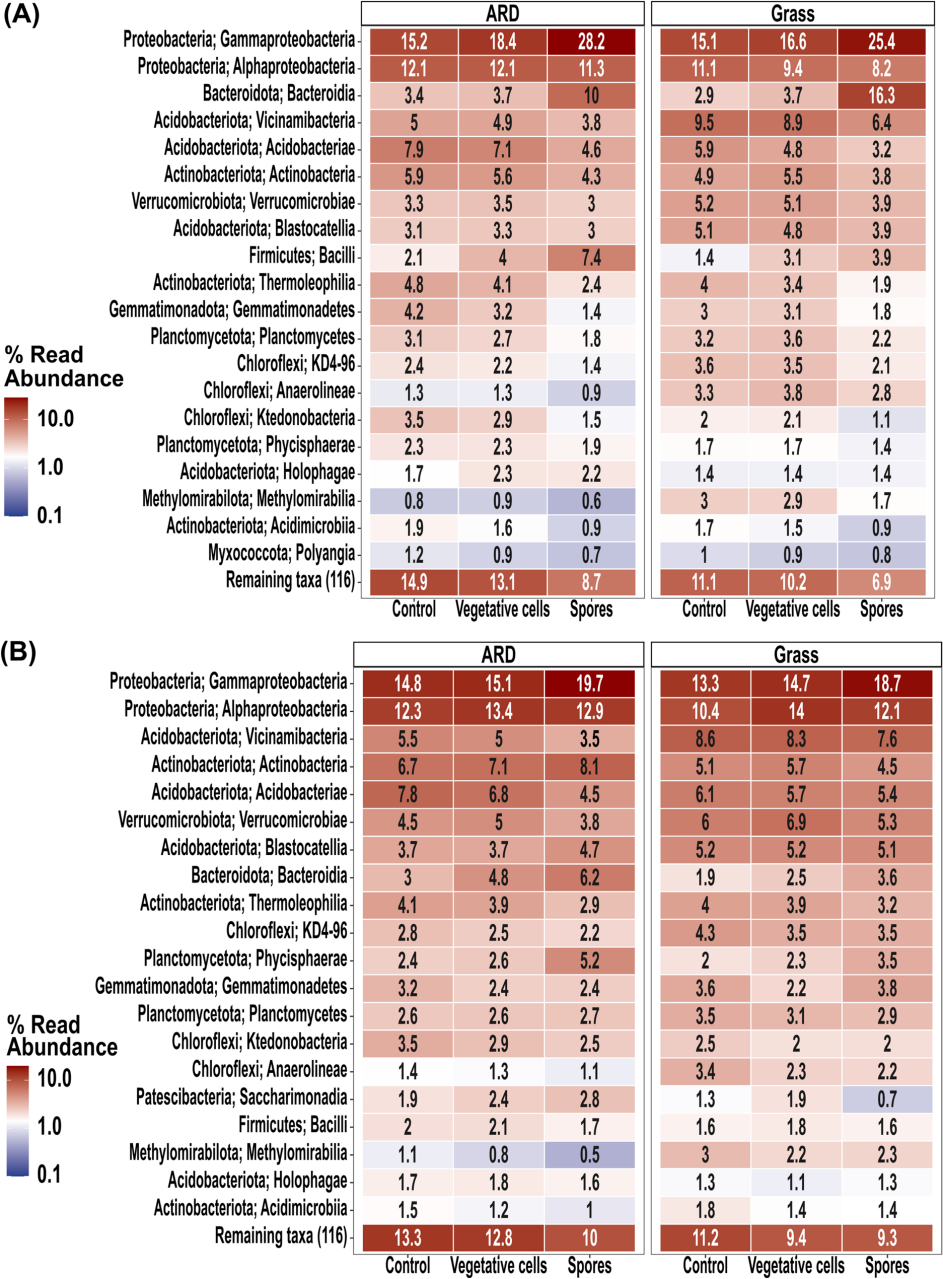
Heatmap representing the relative abundances of the top 20 abundant rhizosphere bacterial classes. Plantlets were grown in ARD or grass soil and inoculated with either vegetative cells or spores of *P. megaterium* B1L5 or remained uninoculated as control, at (**A**) 6 and (**B**) 33 dpi. Values represent average relative abundance of 6 replicates of each treatment. Names composed of numbers and letters represent taxa from groups lacking validly published scientific names, primarily sequenced through environmental studies

Gammaproteobacteria was the most abundant bacterial class in both grass and ARD soils at 6 and 33 dpi, across all treatments, with the highest relative abundances consistently observed in the “Spores” (Fig. 2). In grass soil at 6 dpi, its relative abundance was 15.1% in the “Control,” 16.6% in “Vegetative cells,” and 25.4% in “Spores” (Fig. 2A). At 33 dpi, the values were 13.3%, 14.7% and 18.7%, in “Control”, “Vegetative cells” and “Spores” treatments, respectively (Fig. 2B). In ARD soil, Gammaproteobacteria reached 15.2% in the “Control,” 18.4% in “Vegetative cells,” and 28.2% in “Spores” at 6 dpi, while at 33 dpi it accounted for relative abundance 14.8%, 15.1% and 19.7% in “Control”, “Vegetative cells” and “Spores”, respectively (Fig. 2A and B). At 6 dpi, in both soils, “Spores” treatment displayed high relative abundance of Bacteroidia, compared to both “Control” and “Vegetative cells” (Fig. 2).

In both soils, at 6 dpi *Arthrobacter*, *Massilia*, *Bacillus*, *Candidatus Udaeobacter*, *Sphingomonas*, *Flavobacterium*, *Pseudomons* and *Rhodanobacter* were among the top 20 bacterial genera (Figure S7A). At 6 dpi *Bacillus* displayed higher relative abundance in treatments “Vegetative cells” (2.9% in grass soil and 3.6% in ARD soil) and “Spores” (2.5% in grass soil and 6% in ARD soil), compared to “Control” (1.2% in grass soil and 1.6% in ARD soil) (Figure S7A). In both soils, the relative abundance of *Massilia* was the highest in “Spores”, reaching 3.9% and 6.9% in grass and ARD soils, respectively (Figure S7A). Similarly, *Flavobacterium* exhibited the highest relative abundance in treatment “Spores”, accounting for 7.3% in grass soil and 3.8% in ARD soil (Figure S7A). At 33 dpi *Bacillus*, *Candidatus Udaeobacter*, *Sphinogomonas*, *Arthrobacter* and *Rhodanobacter* were still among the top abundant 20 bacterial genera (Figure S7B).

#### Differentially abundant taxa in the rhizosphere of inoculated plants compared to controls

**Fig. 3:**
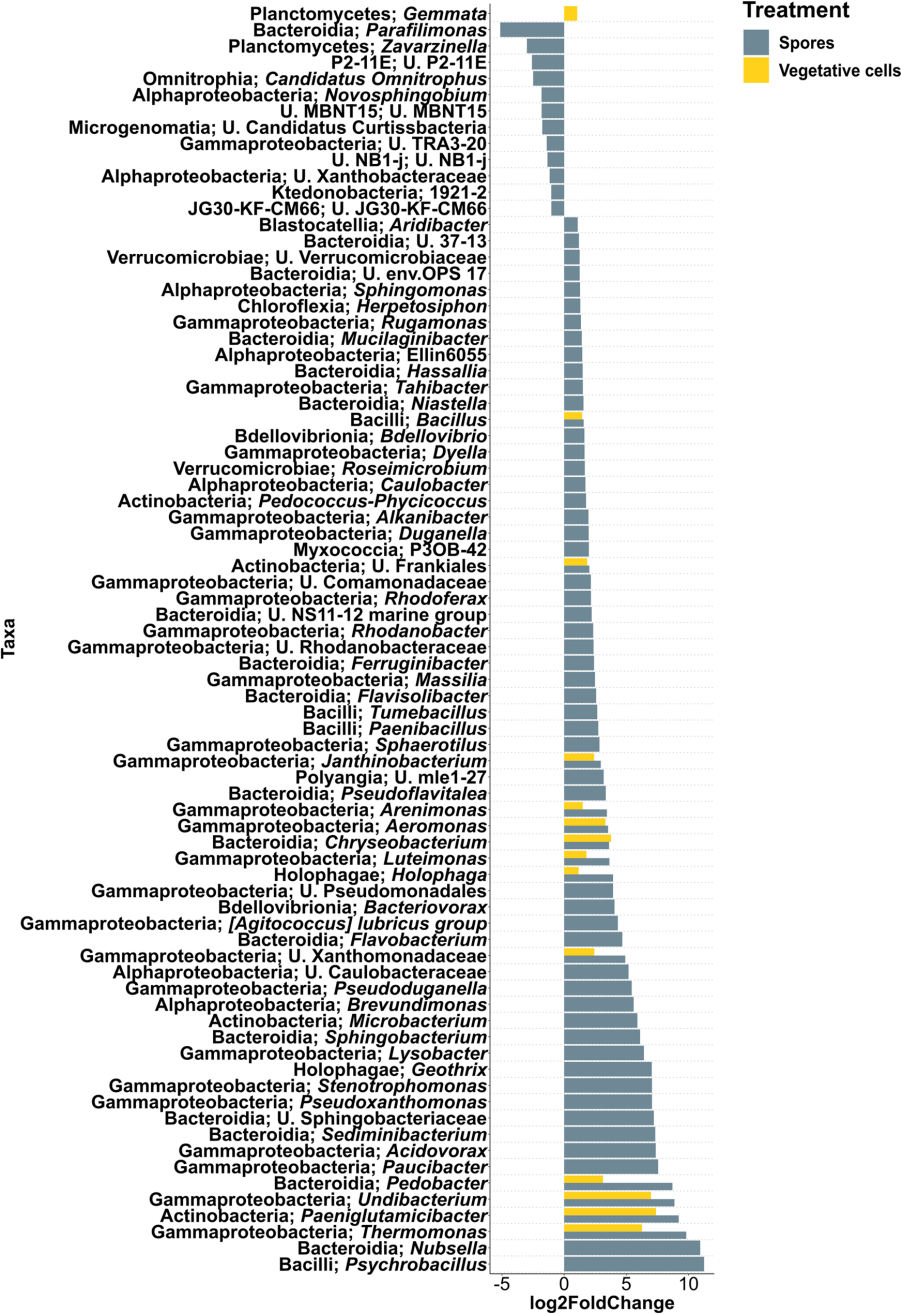
Differential abundance analysis of rhizosphere bacterial genera of apple plantlets grown in grass soil at 6 dpi. Plantlets inoculated with either vegetative cells or spores of *P. megaterium* B1L5 were compared to uninoculated control plantlets. The analysis was performed using R package DESeq2 v.1.42.0. Log2-fold changes are shown on the x-axis. On the y-axis differentially abundant taxa with a *p* value < 0.05 are displayed

**Fig. 4:**
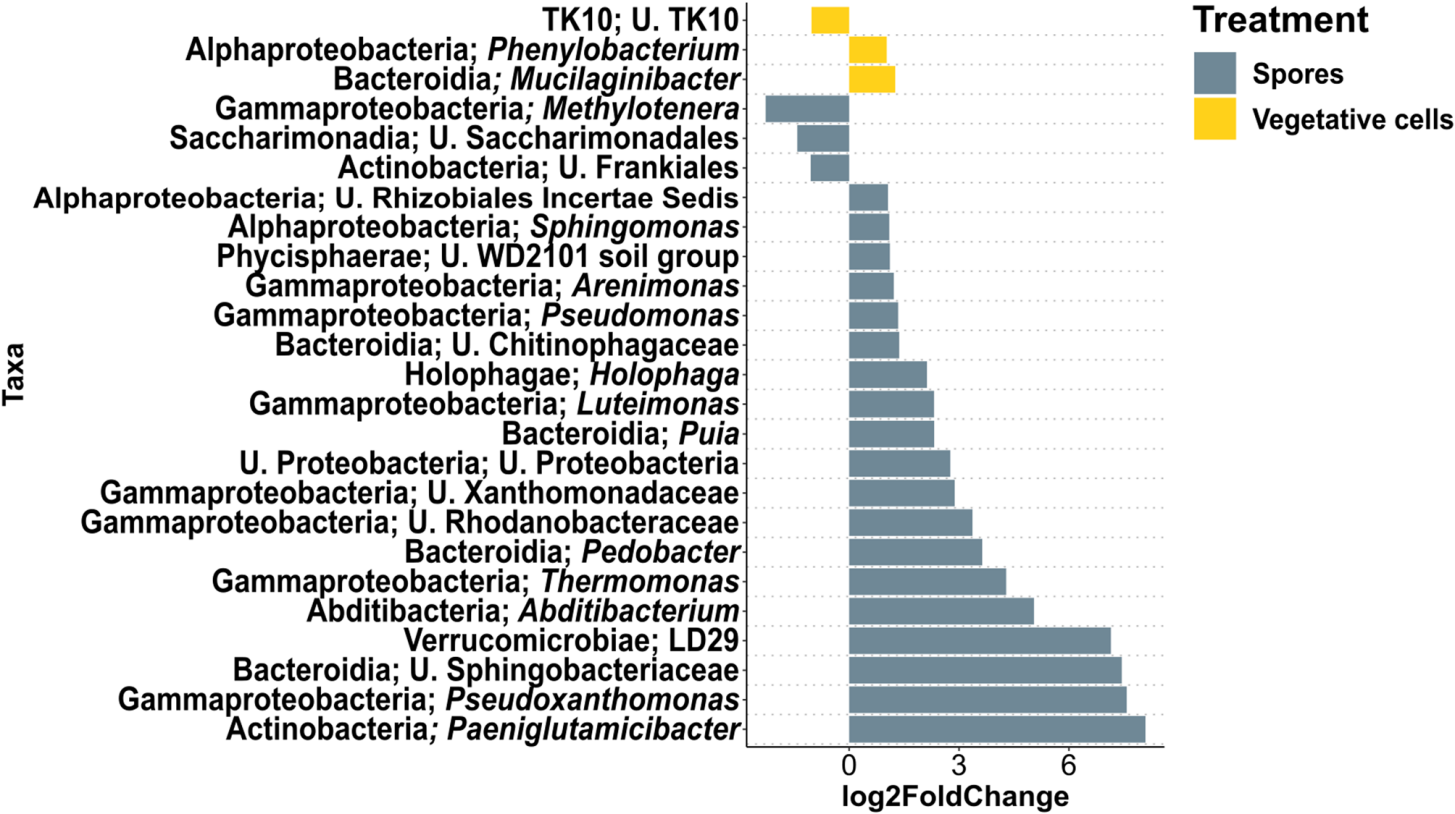
Differential abundance analysis of rhizosphere bacterial genera of apple plantlets grown in grass soil at 33 dpi. Plantlets inoculated with either vegetative cells or spores of *P. megaterium* B1L5 were compared to uninoculated control plantlets. The analysis was performed using R package DESeq2 v.1.42.0. Log2-fold changes are shown on the x-axis. On the y-axis differentially abundant taxa with a *p* value < 0.05 are displayed

In grass soil at 6 dpi, rhizosphere communities in both “Spores” and “Vegetative cells” treatments displayed significantly higher abundances of 13 bacterial genera compared to the uninoculated control, including *Arenimonas*, *Aeromonas*, *Bacillus*, *Luteimonas*, *Pedobacter* and *Janthinobacterium* (Fig. 3). A total of 52 bacterial taxa were significantly enriched in “Spores” treatment compared to the control, among which *Flavobacterium*, *Lysobacter*, *Paenibacillus*, *Sphingobacterium*, *Rhodanobacter*, *Massilia* and *Sphinogomonas* were recorded. Rhizospheres of the “Spores” treatment were significantly depleted in 12 bacterial taxa, where *Parafilimonas* and *Zavarzinella* were the least abundant. *Gemmata* was significantly more abundant only in the vegetative cell treatment. At 33 dpi, 19 bacterial genera were significantly enriched in the rhizosphere of “Spores” treatment, with *Sphingomonas*, *Arenimonas*, *Luteimonas* and *Pedobacter* still among the enriched bacterial genera as at 6 dpi (Fig. 4). *Pseudomonas* was also significantly more abundant in “Spores” treatment, while *Methylotenera*, unidentified *Saccharimonadales*, and unidentified Frankiales were significantly reduced. In “Vegetative cells” treatment, *Phenylobacterium* and *Mucilaginibacter* showed a significantly higher abundance compared to “Control”.

**Fig. 5:**
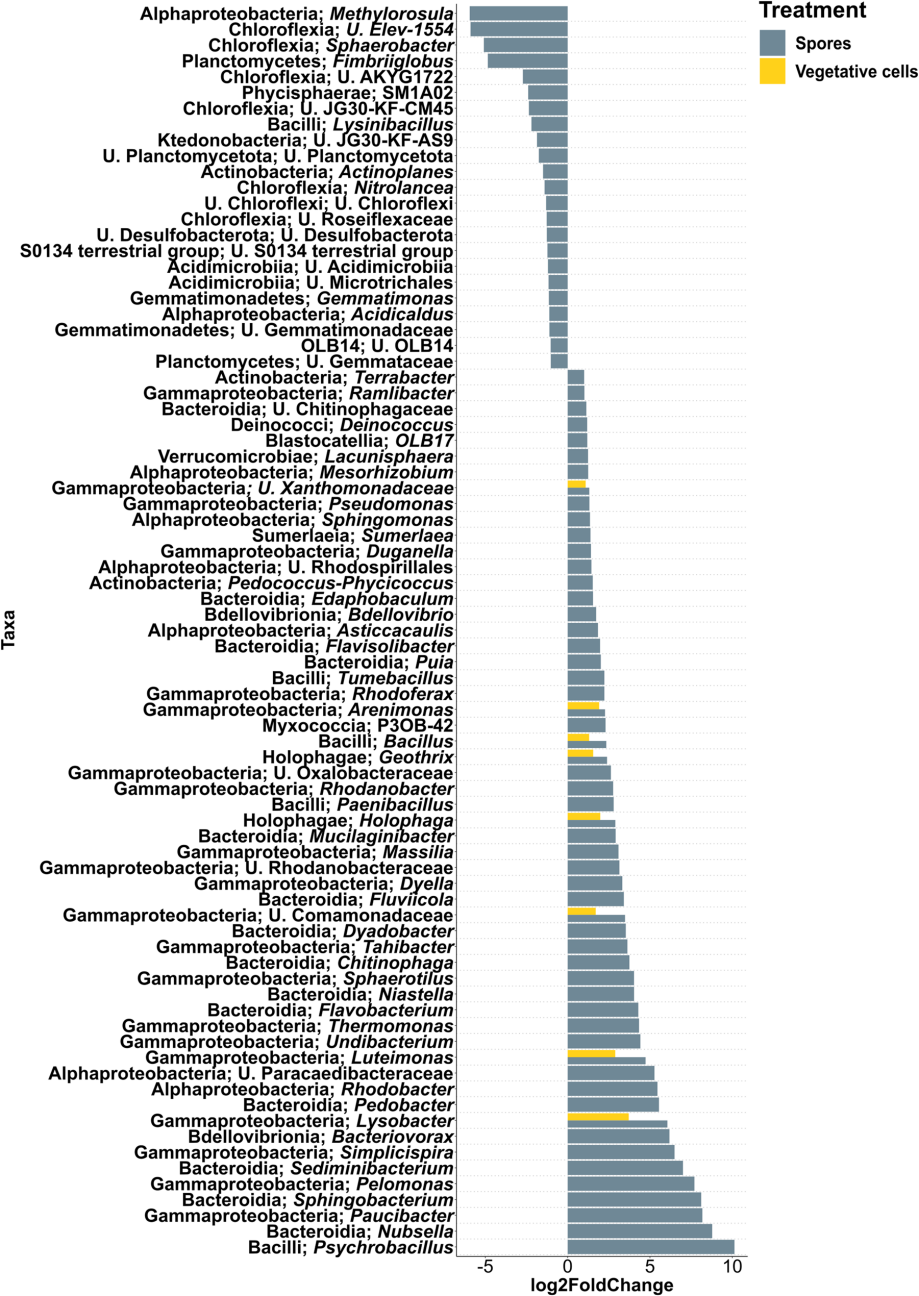
Differential abundance analysis of bacterial genera in the rhizosphere of apple plantlets grown in ARD soil at 6 dpi. Plantlets inoculated with either vegetative cells or spores of *P. megaterium* B1L5 were compared to uninoculated control plantlets. The analysis was performed using R package DESeq2 v.1.42.0. Log2-fold changes are shown on the x-axis. On the y-axis differentially abundant taxa with a *p* value < 0.05 are displayed

**Fig. 6:**
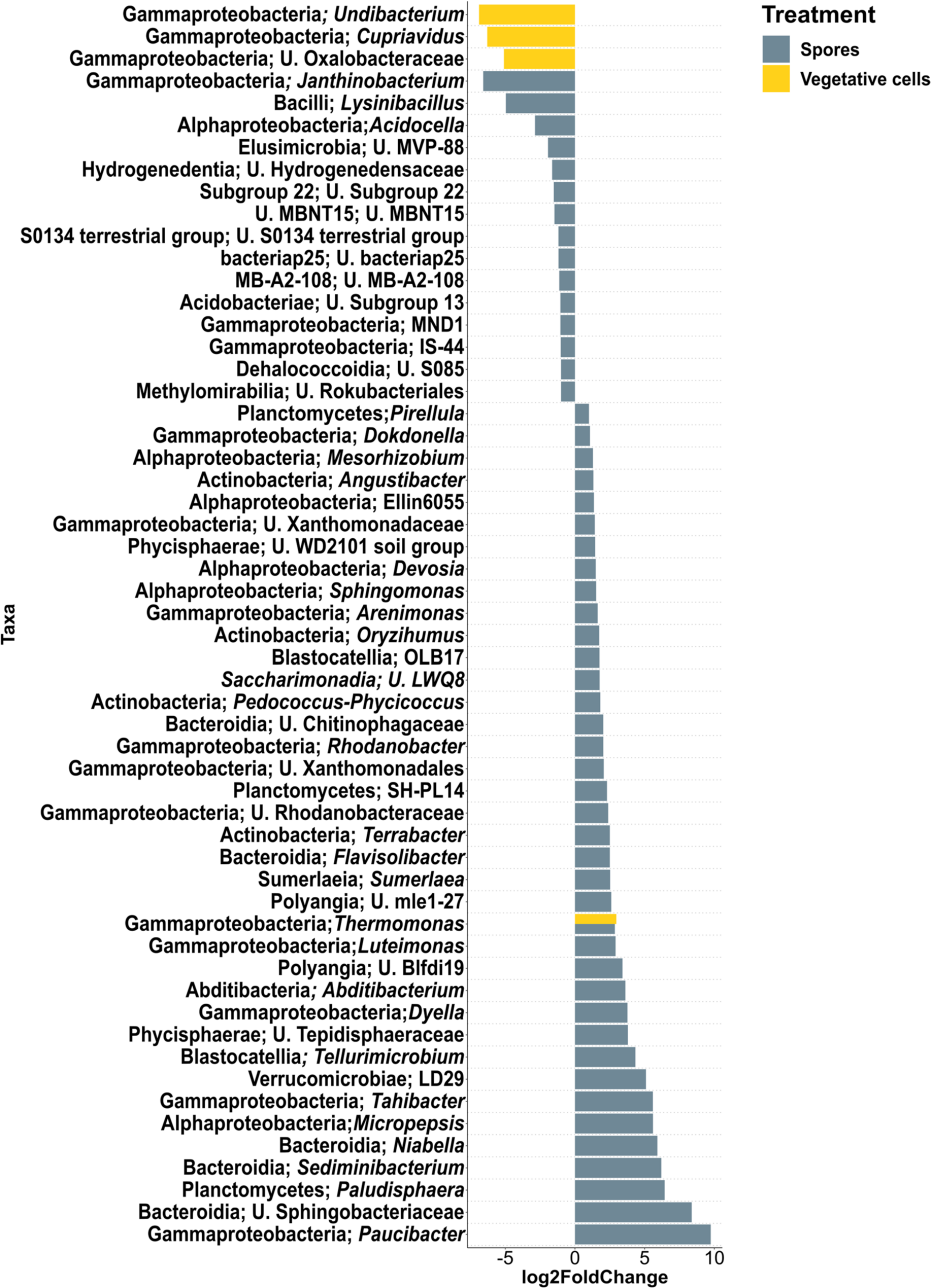
Differential abundance analysis of bacterial genera in the rhizosphere of apple plantlets grown in ARD soil at 33 dpi. Plantlets inoculated with either vegetative cells or spores of *P. megaterium* B1L5 were compared to uninoculated control plantlets. The analysis was performed using R package DESeq2 v.1.42.0. Log2-fold changes are shown on the x-axis. On the y-axis differentially abundant taxa with a *p* value < 0.05 are displayed

In ARD soil at 6 dpi, 8 bacterial taxa were significantly enriched in the rhizosphere of vegetative cells- and spore-inoculated plantlets including *Bacillus*, *Luteimonas* and *Lysobacter* (Fig. 5). Forty-eight bacterial taxa were significantly higher in abundance in “Spores” treatment, among which *Flavobacterium*, *Massilia*, *Paenibacillus*, *Arenimonas*, *Pseudomonas*, *Sphingomonas*, *Sphingobacterium*, *Pedobacter* and *Rhodanobacter* were observed. *Methylorosula*, *Sphaerobacter* and *Fimbriiglobus* were genera with a significantly higher abundance in “Control” compared to “Spores”. At 33 dpi, 37 bacterial taxa were significantly more abundant in “Spores” compared to “Control”, where *Paucibacter*, *Paludisphaera*, *Sediminibacterium*, *Niabella* and *Micropepsis* were among the most highly enriched taxa (Fig. 6). *Sphingomonas*, *Arenimonas*, *Rhodanobacter* and *Luteimonas* remained significantly more abundant in “Spores” treatment. *Janthinobacterium*, *Lysinibacillus* and *Acidocella* were significantly depleted in “Spores”, while *Undibacterium*, *Cuprividus* and unidentified *Oxalobacteraceae* were significantly lower in “Vegetative cells”, compared to “Control”.

In both grass and ARD soil most bacterial genera that were significantly enriched or depleted in the rhizosphere of inoculated plantlets at 33 dpi relative to controls, were not significantly altered over time in uninoculated controls when comparing 33 dpi to 6 dpi. In grass soil, however, temporal changes in the control treatment (33 dpi vs. 6 dpi) included enrichment of unidentified Frankiales and Saccharimonadales, and depletion of *Phenylobacterium* and *Pseudomonas* (Figure S8). In contrast, in the rhizosphere of inoculated plantlets at 33 dpi, these trends were reversed: Frankiales and Saccharimonadales were significantly depleted, while *Phenylobacterium* and *Pseudomonas* were enriched compared to the control. In ARD soil, the abundance of both unidentified Rhodanobacteraceae and *Luteimonas* was significantly higher in “Control” at 33 dpi compared to 6 dpi, and were further enriched in the rhizosphere of spore-inoculated plantlets at 33 dpi (Figure S9).

### Effect of inoculation of *P. megaterium* B1L5 on fungal communities of the rhizosphere

#### Diversity of fungal communities

**Fig. 7:**
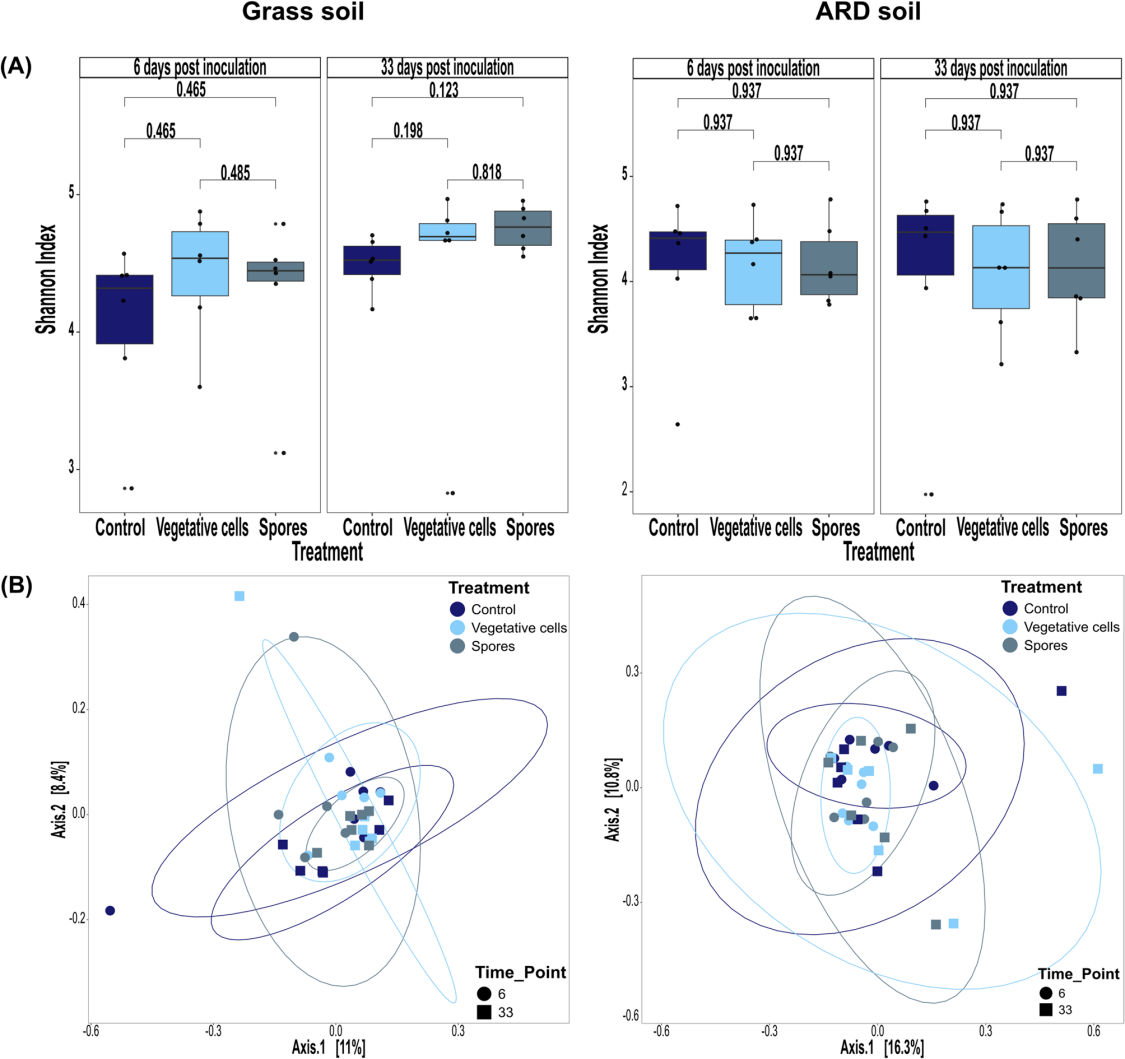
Diversity of rhizosphere fungal communities of apple plantlets grown in grass or ARD soil. Plantlets were inoculated with either vegetative cells or spores of *P. megaterium* B1L5 or remained uninoculated as control and sampled at 6 and 33 dpi. (**A**) Alpha diversity, represented by Shannon index; significance (*p* < 0.05) was determined using the Wilcoxon test, and adjusted for multiple comparisons using the Benjamini-Hochberg (BH) method. (**B**) Beta diversity of rhizosphere fungal communities demonstrated by principal coordinates analysis (PCoA) based on Bray–Curtis distance

Following data processing, 1,839,816 reads were clustered into 5,711 fungal ASVs. Alpha diversity of fungal communities was not significantly affected by the inoculation of vegetative cells or spores, neither in grass nor in ARD soil (Figs. 7A, S10A & S10B). Additionally, the structure of fungal communities in the rhizosphere of both soils (grass or ARD) was not affected by inoculation with vegetative cells or spores, as shown by the clustering patterns in the PCoA (Fig. 7B). This was further supported by the PERMANOVA test, which demonstrated no significant influence of the treatment on fungal community composition (“grass soil: R2 = 0.06, F = 0.99, *P* value = 0.502”, “ARD soil: R2 = 0.047, F = 0.82, *p* value = 0.865”) (Table S7). However, fungal communities of treatments “Control” “Vegetative cells” and “Spores” differed significantly between grass and ARD soils (Figure S11).

#### Taxonomic composition

**Fig. 8:**
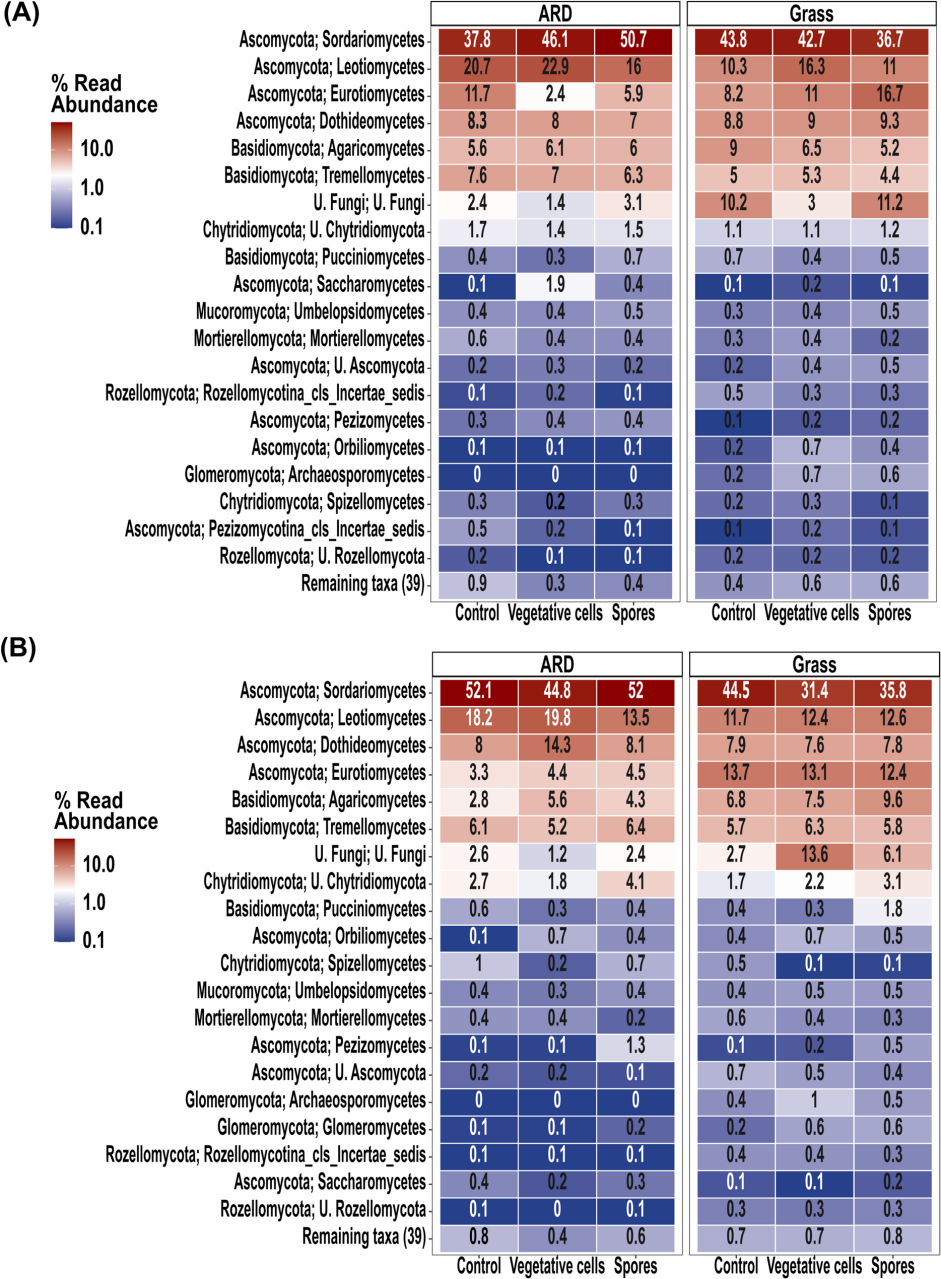
Heatmap representing the relative abundance of top 20 abundant rhizosphere fungal classes. Plantlets were grown in ARD or grass soil and inoculated with either vegetative cells or spores of *P. megaterium* B1L5 or remained uninoculated as control at (**A**) 6 and (**B**) 33 dpi. Values represent average relative abundance of 6 replicates of each treatment. U.’ denotes an unidentified class within this higher taxonomic group. Names composed of numbers and letters represent taxa from groups lacking validly published scientific names, primarily sequenced through environmental studies

In both, grass and ARD soils, fungal communities were dominated by Ascomycota in all treatments (> 50%) at both time points, specifically the class Sordariomycetes (Fig. 8A and B). At 6 and 33 dpi, Archaeosporomycetes (phylum Glomeromycota) was only detected in grass soil, but not in ARD soil (Fig. 8). In ARD soil, *Fusarium* was the most abundant genus, at 6 and 33 dpi (Figures S12A and S12B). In both soils, at 6 and 33 dpi, *Gibellulopsis*, *Hymenoscyphus*, *Cladorrhinum*, *Neocosmospora*, *Saitozyma*, *Solicoccozyma*, *Tetracladium*, *Trichocladium*, *Mycoarthris* and *Ilyonectria*, were among the top 20 genera (Figures S12A and S12B). *Robillarda* was undetected in the rhizosphere of all treatments of grass soil, while *Exophiala* displayed very low relative abundance in ARD soil compared to grass soil in all treatments (Figures S12A and S12B).

#### Differentially abundant taxa in the rhizosphere of inoculated plants compared to controls

**Fig. 9:**
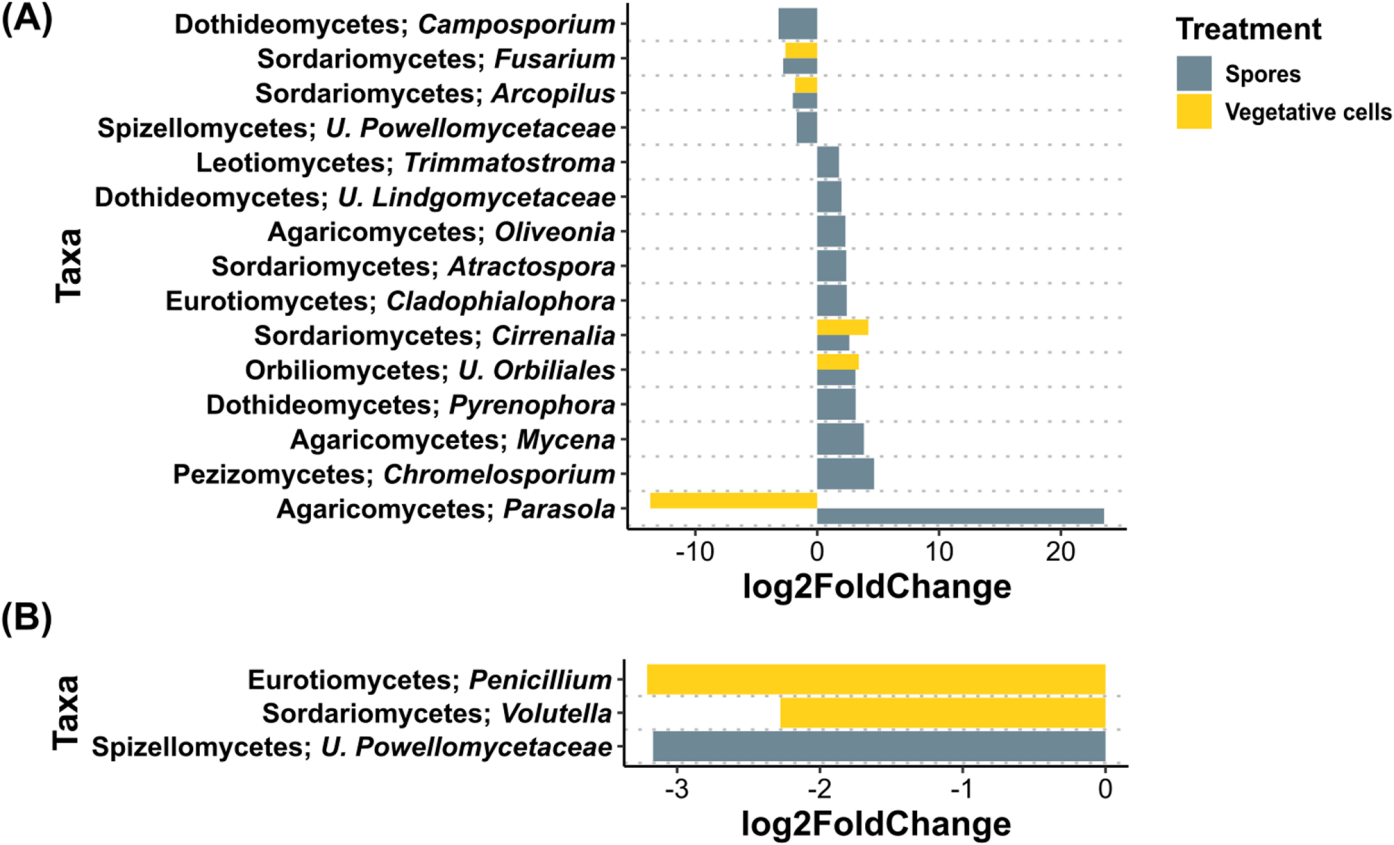
Differential abundance analysis of rhizosphere fungal genera rhizosphere of apple plantlets grown in grass soil. Plantlets inoculated with either vegetative cells or spores of *P. megaterium* B1L5 were compared to uninoculated control plantlets, at (**A**) 6 and (**B**) 33 dpi. The analysis was performed using R package DESeq2 v.1.42.0. Log2-fold changes are shown on the x-axis. On the y-axis differentially abundant taxa with a *p* value < 0.05 are displayed

In contrast to bacterial communities, few fungal genera exhibited significant differential abundances in the rhizosphere of treated plants compared to the controls in both grass and ARD soils at both time points. In grass soil at 6 dpi, *Fusarium* and *Arcopilus* were significantly reduced in treatments “Vegetative cells” and “Spores” compared to “Control”, while *Cirrenalia* and unidentified *Orbiliales* were enriched (Fig. 9A). Genera significantly enriched in “Spores” treatment included *Parasola*, *Chromelosporium*, *Mycena*, *Pyrenophora*, *Cladophialophora*, *Atractospora*, *Oliveonia* and *Trimmatostroma*. *Camposporium* and *Parasola* were significantly higher in “Control” compared to “Spores” and “Vegetative cells” treatments, respectively. At 33dpi, *Penicillium* and *Volutella* were significantly reduced in response to vegetative cells inoculation, while U. Powellomycetaceae was significantly lower after spore inoculation (Fig. 9B).

**Fig. 10:**
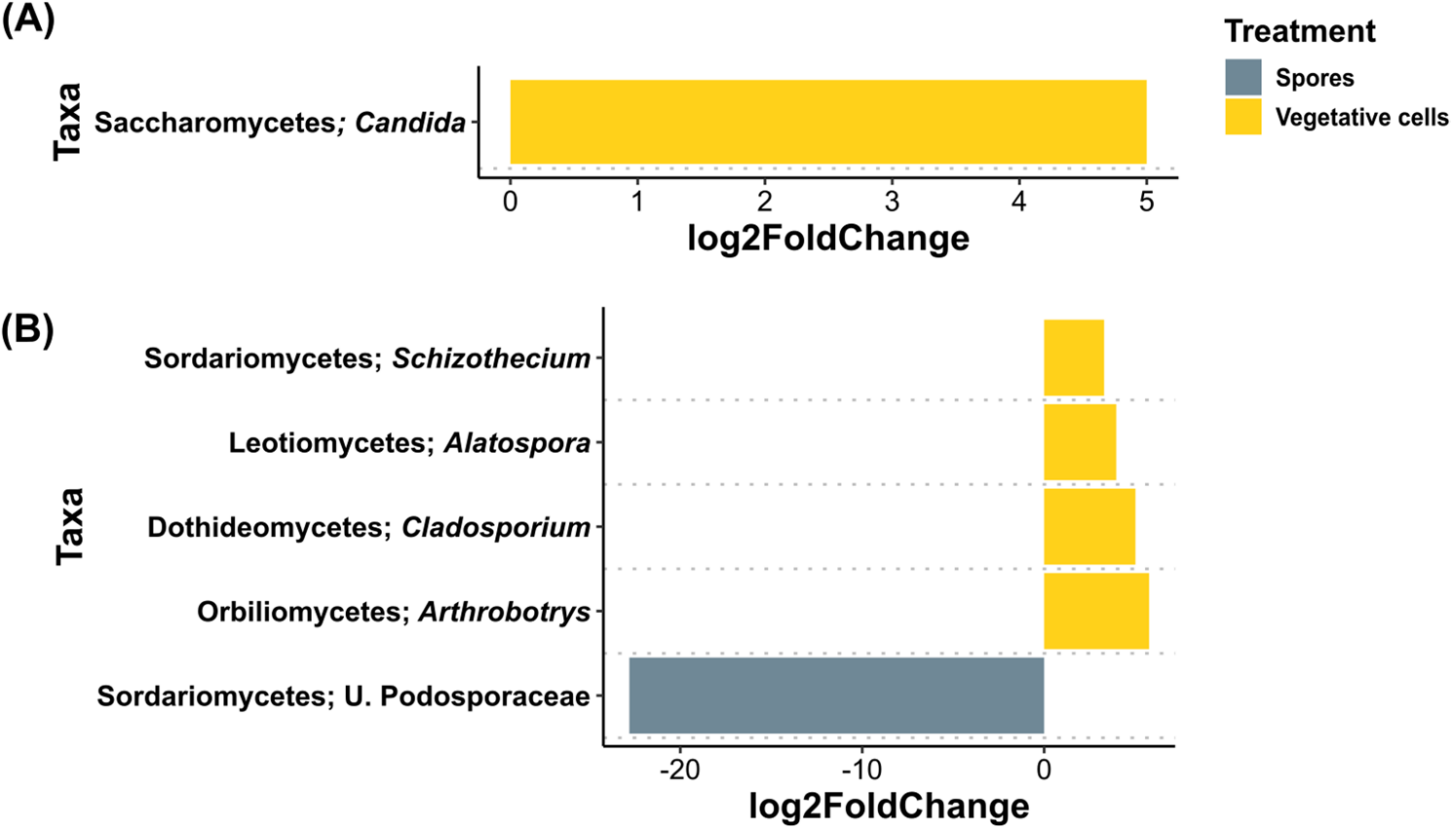
Differential abundance analysis of rhizosphere fungal genera rhizosphere of apple plantlets grown in ARD soil. Plantlets inoculated with either vegetative cells or spores of *P. megaterium* B1L5 were compared to uninoculated control plantlets at (**A**) 6 and (**B**) 33 dpi. The analysis was performed using R package DESeq2 v.1.42.0. Log2-fold changes are shown on the x-axis. On the y-axis differentially abundant taxa with a *p* value < 0.05 are displayed

In ARD soil at 6 dpi, *Candida* was the only genus significantly differentially abundant and significantly lower in “Vegetative cells” treatment (Figs. 10A). At 33 dpi, *Schizothecium*, *Alatospora*, *Cladosporium* and *Arthrobotrys* were significantly higher in the “Vegetative cells” treatment, while unidentified *Podosporaceae* was significantly lower in “Spores” treatment compared to the “Control” (Figs. 10B).

In ARD soil, most fungal genera enriched or depleted in inoculated rhizospheres at 33 dpi were not differentially abundant in uninoculated controls when comparing 33 dpi to 6 dpi. The only exceptions were *Alatospora* and *Cladosporium*, which were significantly depleted in ARD control plants at 33 dpi relative to 6 dpi, while being enriched in inoculated plants at 33 dpi (Figure S13). In grass soil, no genera were significantly differentially abundant between 33 and 6 dpi in uninoculated control plantlets.

## Discussion

We examined the colonization and establishment of the GFP-labelled strain *P. megaterium* B1L5 in the root-associated environment of apple plants and its potential role in mitigation of ARD symptoms. Additionally, we investigated its influence on rhizosphere microbial communities, with a particular emphasis on how modulation of microbial communities could potentially support plant health, especially in an ARD-affected soil. We hypothesized that *P. megaterium* B1L5 would effectively colonize apple roots (H1), enhance plant growth and reduce ARD symptoms (H2). Additionally, we anticipated that it will alter rhizosphere bacterial and fungal communities (H3).

### Colonization dynamics of *P. megaterium* B1L5 in apple roots

We used a GFP-labelled mutant of *P. megaterium* B1 designated as *P. megaterium* B1L5 to visually track the colonization of roots in addition to a qPCR system targeting the *gfp* gene for quantitative assessment. At 6 dpi *P. megaterium* B1L5 displayed successful colonization of apple roots, irrespective of whether it was introduced as vegetative cells or spores, and whether the plantlets were grown in grass or ARD soil. This confirmed our previous results [47] that *Priestia megaterium* B1 isolated from apple roots has the potential for successful root colonization. However, by 33 dpi, colonization by strain B1L5 was no longer detectable, indicating a lack of long-term persistence in the apple root environment. The failure to persist could be attributed to the inability of strain B1L5 to adapt to the compounds exuded by the apple roots over time. In response to biotic or abiotic elicitors, apple plantlets tend to accumulate phytoalexins in their roots and exude them in the surrounding rhizosphere [8, 13, 82]. Phytoalexins have been known for their antimicrobial activity and potential role in plant defense against plant pathogens [8, 82]. However, an accumulation of phytoalexins in apple roots has also been reported in response to inoculation of the plant growth-promoting bacterial strains *Bacillus velezensis* FZB42 and *Pseudomonas* sp. RU47 in plantlets grown in healthy grass soil [22]. This indicates a plant defense reaction of apple to microbes regardless of being beneficial or pathogenic. Therefore, it is possible that the proliferation and persistence of strain *P. megaterium* B1L5 was hindered by phytoalexins present in the roots and/or in the rhizosphere.

Another possible explanation for the transient establishment of strain B1L5 in the rhizosphere is that, over time, it may have been outcompeted by the established microbial communities associated with apple roots. Studies employing diversity gradient experiments showed that survival of microbial invaders could be impeded by resource competition and diversity of resident microbial communities [3–5]. An additional explanation for the limited establishment could be that it is not a true endophyte but rather a colonizer of the rhizosphere or rhizoplane. Despite being isolated from surface-sterilized apple roots, it is possible that it wasn’t actively colonizing root internal tissue and its spore-forming ability may have enabled it to survive the sterilization process. The observed colonization pattern of *P. megaterium* B1L5, restricted to the root surface and the outermost root layers, suggests a passive colonization mode possibly aided by the high inoculation concentration and root entry through natural openings as lateral root emerging points or surface cracks, resulting in limited penetration of root layers [83, 84]. This contrasts active colonization where endophytes employ specific mechanisms such as motility, lipopolysaccharides, quorum sensing and production of cell wall-degrading enzymes to penetrate deeper into root tissues [83]. Active colonizers, compared to passive ones, can establish long-term association with plant roots as they reside within plant tissues, thus being protected from environmental fluctuations and microbial competition. While *P. megaterium* B1L5 possesses genetic features, suggesting active colonization potential, including genes related to motility and carbohydrate-active enzymes [47], its inability to persist suggests that host factors, resident microbial communities or environmental conditions may have resulted in its limited colonization efficiency.

### ARD occurrence and potential role of *P. megaterium* B1L5 in ARD mitigation

The significantly higher percentage of blackened root tips in plants grown in ARD-affected soil compared to grass soil shows the presence of ARD, as root tip necrosis is a typical ARD symptom [60]. The absence of growth reduction in ARD-affected soil relative to ARD-unaffected grass soil could be attributed to the short duration of the experiment where the early symptoms of a damaged root system did not yet translate into significant growth effects. The ARD soil used in our study was collected from the Heidgraben site, which is known for its history of apple monoculture and well documented ability to induce ARD symptoms [53, 54]. However, the extent to which ARD reduced plant growth varied across the studies when comparing ARD-affected soil to ARD-unaffected natural soils such as grass soil [53, 54]. Such variability in ARD-related growth suppression may be driven by fluctuations in the abundance and the relative contribution of the pathogen complex to the disease, which can vary across years, geographical regions and orchards [15].

While no immediate growth enhancement was observed in plants grown in ARD-affected soil following inoculation with *P. megaterium* B1L5, the notable reduction in blackened root tips highlights its potential to reduce ARD-related root symptoms, suggesting that over a longer period beneficial effects on plant growth may become more apparent. This aligns with previous studies demonstrating the biocontrol capacity of *Priestia* (formerly *Bacillus*) strains and their ability to mitigate disease symptoms [44, 45, 85–88]. Notably, genome mining of the wild-type strain *P. megaterium* B1 using antiSMASH revealed the presence of a biosynthetic gene cluster encoding surfactins [47], cyclic lipopeptides known for their antimicrobial activity against plant pathogens, primarily through disruption of pathogen membranes. In addition, surfactins have been reported to elicit induced systemic resistance in plants [89–91]. Both mechanisms could plausibly contribute to the observed symptom reduction. Other indirect modes of action such as interference with quorum sensing (“quorum quenching”) and suppression of pathogen virulence may also play a role [92, 93]. However, to clarify the underlying mechanisms, future studies should investigate the *in planta* expression and functional activity of surfactin biosynthetic genes, monitor pathogen activity and virulence and assess host immune responses such as those associated with induced systemic resistance.

Enhancing the persistence of *P. megaterium* B1L5 in the root environment could further amplify its beneficial effects, as longer establishment in the root-associated environment can increase chances of beneficial inoculants to exert their growth-promoting effects [2]. Recurrent inoculation or carrier-based formulations could extend its presence in apple roots-associated environments [94, 95]. Moreover, its incorporation into a synergistic consortium of plant growth-promoting microbes could enhance its functionality, as studies showed that microbial consortia are more effective at promoting plant growth than individual strains [96, 97].

### Influence of *P. megaterium* B1L5 on apple rhizosphere microbiome

While *P. megaterium* B1L5 did not detectably persist, the rhizosphere bacterial communities of apple plantlets grown in grass or ARD soil were lastingly changed, highlighting the underexplored effects of transient and apparently unsuccessfully established invaders on microbial communities [98]. Beta diversity and PERMANOVA analyses indicated that the composition of rhizosphere bacterial communities grown in grass or ARD soil, were significantly affected after inoculation of *P. megaterium* B1L5. Additionally, the inoculation of *P. megaterium* B1L5 significantly enriched the rhizosphere of apple plantlets grown in grass and ARD soils, with potential beneficial bacterial genera including *Luteimonas*, *Lysobacter*, *Pseudomonas*, *Sphingomonas*, *Paenibacillus*, *Flavobacterium*, *Sphingobacterium*, *Rhodanobacter* and *Pedobacter. Luteimonas* has been recognized for enhancing plant resistance to pathogens [99]. Similarly, *Lysobacter* isolates were reported for exhibiting antimicrobial activity against plant pathogens [100, 101]. *Pseudomonas* is a genus well known for its members with plant growth-promoting capabilities [102]. Some isolates of *Pseudomonas* showed the ability to suppress the nematode *Pratylenchus penetrans*, which is a potential contributor to ARD in apple orchards [103, 104]. Nicola et al. (2017) demonstrated a positive correlation between the abundance of *Pseudomonas* and growth of plants in replanted soil [24]. Additionally, *Sphingomonas* has been reported to produce plant growth-promoting metabolites, such as auxin, siderophores and gibberellins, possibly contributing to plant growth [105, 106]. It has been also known for modulating plant hormones including abscisic acid, salicylic acid and jasmonic acid [105], as well as producing antioxidant enzymes [107] which contribute to enhancing plant resistance. Likewise, *Sphingobacterium* was reported to enhance plant antioxidant capacity and improve its tolerance to stress [108]. *Paenibacillus* and *Flavobacterium* contain species known for promoting plant growth and exhibiting antimicrobial activity against plant pathogens [109–112]. *Rhodanobacter* was also reported to exhibit antifungal properties [113], while *Pedobacter* displayed plant growth-promoting potential [114]. The enrichment of bacterial genera known for their potential plant-beneficial traits suggests a possible shift toward a more beneficial microbial community. In ARD soil, where plant-induced microbial imbalances contribute to the disease development [15–17], this kind of shift could be particularly meaningful. While bacterial inoculants have been extensively investigated as a potential approach to mitigate ARD [29–34], investigating their influence on rhizosphere and root-associated microbial communities remains limited [22, 32, 33]. Our results help to fill this gap by showing that even a transient inoculant such as *P. megaterium* B1L5 can enrich potentially helpful microbes, adding to our understanding of how inoculants could affect rhizosphere communities.

In contrast to bacterial communities, fungal communities were less affected. The minimal influence of microbial inoculation on the composition of fungal communities, compared to that of bacterial communities, was previously observed [115]. However, inoculation with vegetative cells or spores of *P. megaterium* B1L5 significantly reduced *Fusarium* in the grass soil, while this effect was not observed in ARD soil. These contrasting responses suggest that the interaction between *P. megaterium* B1L5 and the resident fungal communities could depend on soil health. Balbín-Suárez et al. (2020, 2021) highlighted the dysbiosis, or disruption, of root-associated microbial communities in ARD soil compared to grass soil [16, 17], while Radl et al. (2019) found a higher abundance of genes associated with antibiotic biosynthesis and stress sensing in the ARD rhizosphere, indicating the antagonistic and stressful nature of this environment [23]. This disrupted and stressful soil environment possibly hindered *P. megaterium* B1L5’s potential to effectively limit pathogens such as *Fusarium*. In contrast, the healthier and more balanced microbial community in grass soil likely facilitated *P. megaterium* B1L5 in suppressing pathogens like *Fusarium*.

While changes in rhizosphere microbial communities are expected over time as plants grow and develop [116–118], our findings demonstrated that the detected shifts in rhizosphere microbial communities of inoculated plantlets were largely driven by treatment rather than temporal changes. Most genera enriched or depleted in the rhizosphere of plantlets inoculated by *P. megaterium* B1L5 at 33 dpi did not exhibit similar shifts in uninoculated controls over time, including bacterial genera with potential plant-beneficial traits. For example, in grass soil, *Pseudomonas*, *Luteimonas*, *Pedobacter*, and *Sphingomonas* were significantly enriched in spore-inoculated plantlets at 33 dpi, while none of these genera showed enrichment in controls over time; notably, *Pseudomonas* was significantly depleted. Similarly, in ARD soil, *Sphingomonas* and *Rhodanobacter* were enriched only in treatment “Spores” compared to “Control” at 33 dpi, while their abundance did not significantly differ in “Control” 33 dpi vs. 6 dpi. Although the abundance of *Luteimonas* increased in “Control” over time, its enrichment was significantly more pronounced in the rhizosphere of spore-inoculated plants at 33 dpi, suggesting that inoculation amplified their natural temporal abundance. These patterns indicate that the observed microbial shifts were primarily treatment-specific and not only mediated by plant development or rhizosphere microbial succession.

## Conclusions and outlook

Our study showed that *P. megaterium* B1L5, whether applied as vegetative cells or spores, was able to transiently colonize the roots of apple plantlets grown in both grass soil (without a history of apple cultivation) and ARD soil, demonstrating its capacity to colonize roots across different soil environments. Notably, inoculated plants grown in ARD soil exhibited a lower degree of root tip blackening, suggesting that *P. megaterium* B1L5 may help reduce early-stage root damage and thus contribute to disease mitigation. Additionally, *P. megaterium* B1L5 had a significant effect on rhizosphere bacterial communities, particularly enriching potential plant-beneficial bacterial taxa. Importantly, most bacterial and fungal taxa enriched or depleted in inoculated plants at 33 dpi were not significantly differentially abundant in uninoculated controls over time (33 dpi vs. 6 dpi), highlighting that the observed microbial shifts were a specific response to inoculation rather than natural plant development and temporal microbial succession.

Future work should involve longer-term experiments to determine whether early reductions in ARD root-related symptoms and positive modulation of rhizosphere microbial communities can translate into improved health of apple plants, particularly during the critical first vegetation period after planting. Additionally, enhancing the persistence of *P. megaterium* B1 in the apple root environment will be crucial to magnify its potential for mitigating ARD-related root symptoms and promoting plant growth. Approaches such as carrier-based formulations, repeated applications, or integration into synergistic microbial consortia could be explored to improve its survival and effectiveness over time.

## Supplementary Information

Below is the link to the electronic supplementary material.

Supplementary Material 1

## Data Availability

Raw reads are deposited in the National Center for Biotechnology (NCBI) under the BioProject PRJNA700828. The 16 S rRNA gene sequencing reads are available under accession numbers SRX26424303 to SRX26424386 and the ITS sequencing reads are deposited under accession numbers SRX26437416 to SRX26437498.
