# Supplementary material for: Transient colonization by Priestia megaterium B1L5 alters the structure of the rhizosphere microbiome towards potential plant beneficial bacterial groups in apple plantlets
Source: Environ Microbiome. 2025 Aug 13;20:104. doi: 10.1186/s40793-025-00762-x (PMC12344982; doi:10.1186/s40793-025-00762-x)

**Supplementary file for the manuscript “Transient colonization by *Priestia megaterium* B1L5 alters the structure of the rhizosphere microbiome towards potential plant beneficial bacterial groups in apple plantlets”**

Fatma M. Mahmoud^a,h^#, Holger Edelmann^b^, Yang Si^c,d^, Lea Endrejat^a^, Karin Pritsch^e^, Caroline Gutjahr^c,d^, Armin Ehrenreich^b^, Traud Winkelmann^f^, Jana Barbro Winkler^e^, Jörg‑Peter Schnitzler^e^, Michael Schloter^a,g^

^a^ Research Unit for Comparative Microbiome Analysis, Helmholtz Munich, German Research Center for Environmental Health, Neuherberg, Germany

^b^ Chair of Microbiology, TUM School of Life Sciences, Technical University of Munich, Freising, Germany

^c^ Plant Genetics, TUM School of Life Sciences, Technical University of Munich, Freising, Germany

^d^ Max-Planck-Institute of Molecular Plant Physiology, Potsdam-Golm, Germany

^e^ Research Unit for Environmental Simulations, Helmholtz Munich, German Research Center for Environmental Health, Neuherberg, Germany

^f^ Institute of Plant Genetics, Leibniz University Hannover, Hanover, Germany

^g^ Chair of Environmental Microbiology, TUM School of Life Sciences, Technical University of Munich, Freising, Germany

^h^ Botany and Microbiology Department, Faculty of Science, Suez Canal University, Ismailia, Egypt

#corresponding author: Fatma M. Mahmoud

**Supplementary materials and methods**

**Design of qPCR system to quantify *gfp* gene copy number in root samples**

An initial qPCR attempt to amplify the full-length *gfp* fragment (741 bp) resulted in low efficiency. Therefore a new forward primer “sf-GFP-F-new, TCAACAGCCACAACGTGTAT”, was designed using the NCBI Primer-BLAST tool, paired with the reverse primer sf-GFP-R “ATTTATACAGTTCATCCATACCATG” targeting a 282 bp amplicon. The specificity of the primers was validated using the NCBI (nt) database to ensure accurate amplification of the intended sequence. Plasmid standards for qPCR were prepared as follows. Partial *gfp* gene was amplified using a conventional PCR reaction comprising of 2 µl of genomic DNA of *P. megaterium* B1L5, 0.5 µl of each of sf-GFP-F-new and sf-GFP-R primers (10 μM), 2.5 μL of 3% BSA, 12.5 μL of NEBNext High-Fidelity 2x PCR Master Mix (New England Biolabs, Frankfurt am Main, Germany), and 7 µl DEPC-treated water. Amplification started with initial denaturation at 98 °C for 30 seconds, followed by 30 cycles of denaturation at 98 °C for 10 seconds, annealing at 60 °C for 30 seconds and extension at 72 °C for 30 seconds and finalized by final extension step at 72 °C for 5 minutes. Success of amplification and size of PCR product was checked using gel electrophoresis. The amplified fragment was cloned in One Shot® TOP10 Chemically Competent *E. coli* using Zero Blunt® PCR Cloning Kit (Invitrogen by life technologies, CA, USA) following the manufacturer’s protocol. Plasmid incorporating *gfp* partial gene was extracted using NucleoSpin™ Plasmid (NoLid) kit (Macherey-Nagel™, Dueren, Germany). Concentration of the extracted plasmid was measured using Qubit^TM^ 4 Fluorometer (Thermo Fisher Scientific, Singapore). The sequence of the inserted fragment was verified using Sanger sequencing performed at Eurofins (Eurofinsgenomics.eu). Number of gene copies (molecules)/ µl in the plasmid standard was quantified according to this equation (1):

DNA (copy) = 6.02 × 10^23^ (copy/mol) × DNA amount (g)

DNA length (db) × 660 (g/mol/db)

For quantification of gene copy number of partial *gfp* gene in the root samples, the qPCR reaction mixture included 12.5 μl Power SYBR™ Green PCR Master Mix (Applied Biosystems by Thermo Fisher Scientific, Darmstadt, Germany), 0.5 μl 3% BSA, 0.5 μl of each of forward and reverse primer, 2 μl of DNA template or plasmid standard and 9 μl DEPC-treated water. The amplification was carried out using the 7300 Real-Time PCR System (Applied Biosystems by Thermo Fisher Scientific, Darmstadt, Germany) under the following conditions: an initial denaturation step at 95°C for 10 minutes, followed by 40 cycles of 95°C for 10 seconds, 60°C for 30 seconds, and 72°C for 60 seconds and finalized by a dissociation phase at 95°C for 15 seconds, 60°C for 30 seconds, and 95°C for 15 seconds. A standard series ranging from 4.18E+08 to 4.18E+01 gene copies μl^−1^ was employed, where amplification was detected till concentration 4.18E+01 gene copies μl^−1^. Negative controls were included, by replacing DNA with 2 μl of DEPC-treated water. The resulting qPCR data were processed using the 7300 System SDS Software v1.3.1.21 (Applied Biosystems). The qPCR efficiency was determined using the formula Eff = [10^(−1/slope)^ − 1] (2) and estimated as 83 % while r^2^ was 0.99. Melting curves were used to check the specificity of the amplified products. Additionally, they were also checked on 1.5% agarose gels.

**Amplicon sequencing and library preparation for rhizosphere bacterial and fungal community analysis**

For analysis of the bacterial community the PCR reaction mixtures consisted of 15 ng of DNA, 0.5 μL of each primer (10 μM), 2.5 μL of 3% BSA, 12.5 μL of NEBNext High-Fidelity 2x PCR Master Mix (New England Biolabs, Frankfurt am Main, Germany), and DEPC-treated water to bring the total volume to 25 μL. The amplification protocol for the 16S rRNA gene began with an initial denaturation at 98 °C for 1 minute, followed by 30 cycles of 98 °C for 10 seconds, 55°C for 30 seconds, and 72 °C for 30 seconds, with a final extension at 72 °C for 5 minutes. PCR products were purified using a Pipetting Robot Biomek4000 (Beckman Coulter, Krefeld, Germany) and MagSi-NGS Plus Beads XP (Steinbrenner, Wiesenbach, Germany), following the manufacturer’s instructions. Indexing PCR was carried out with a reaction mixture containing 10 ng of the purified amplicon, 2.5 μL of each indexing primer (Nextera® XT Index Kit v2 Set C, Illumina, San Diego, CA, USA), 12.5 μL of NEBNext High-Fidelity 2x PCR Master Mix, and DEPC-treated water to adjust the volume to 25 μL. The amplicons were then purified as described above. The quantity and quality of the amplicons were assessed as previously mentioned. The library was diluted to a concentration of 4 nM and pooled equimolarly. Paired-end sequencing was performed on the MiSeq® system (Illumina, San Diego, CA, USA) using the MiSeq® Reagent Kit v3 (600 cycles). For fungal community analysis, the PCR reaction mixtures contained 15 ng of DNA, 0.5 μL of each primer (10 μM), 2.5 μL of 3% BSA, 12.5 μL of NEBNext High-Fidelity 2x PCR Master Mix, and DEPC-treated water to a final volume of 25 μL. The ITS gene amplification protocol began with an initial denaturation at 95°C for 15 minutes, followed by 30 cycles of 95 °C for 30 seconds, 55 °C for 30 seconds, and 72 °C for 60 seconds, ending with a final extension at 72 °C for 10 minutes. Libraries were prepared in the same manner as for the 16S rRNA gene.

**References**

1. Lee C, Kim J, Shin SG, Hwang S. 2006. Absolute and relative QPCR quantification of plasmid copy number in Escherichia coli. J Biotechnol 123:273–280.

2. Töwe S, Albert A, Kleineidam K, Brankatschk R, Dümig A, Welzl G, Munch JC, Zeyer J, Schloter M. 2010. Abundance of Microbes Involved in Nitrogen Transformation in the Rhizosphere of Leucanthemopsis alpina (L.) Heywood Grown in Soils from Different Sites of the Damma Glacier Forefield. Microb Ecol 60:762–770.

**Supplementary tables**

**Table S1:** Conditions for amplification of the DNA fragments for Gibson Assembly

| Nr. | fragment name | primer | template |
| --- | --- | --- | --- |
| 1 | flank1 | A, B | *P. megaterium* B1 |
| 2 | sfGFP-His | C, D | pBACOV-sfGFP |
| 3 | flank2 | G, H | *P. megaterium* B1 |
| 4 | backbone | E, F | pKVM4 |
| 5 | P-groES | O, P | *B. subtilis* (DSM402) |

**Table S2:** Used DNA oligonucleotides

|  | primer name | nucleotide sequence [5‘🡪 3‘] |
| --- | --- | --- |
| A | 00355_Flank1_fwd | GTTTTGGTCGTAGAGCACACGGTT TTATTCGCGGAATTGAAAGC |
| B | 00355-Flank1_sfGFP_rev | CATTCTACAAACTGCATAACTAT CTGCTCACCGTATTAAAGAATC |
| C | sfGFP_fwd_short | ATGAGCAAAGGTGAAGAACTG |
| D | 00355-sfGFP-His6_r | ATTTCTTTGATTCTTTAATACGGTGAGCAG ATAGTTATGCAGTTTGTAGAATGC |
| E | pKVM4-BB-rev | CTTTATTTAACCTGCTTTCAATTCCGCGAATAA AACCGTGTGCTCTACGAC |
| F | pKVM4_BB_fwd | CAATCGTATCAACAAATTTCGGCTTGC AACAGCGTACAGACGATTTAG |
| G | 00355_Flank2_fwd_short | GAGAAACCAGCTGTTAATCAC |
| H | 00355_Flank2_rev | CATCTCTAAATCGTCTGTACGCTGTT GCAAGCCGAAATTTGTTGATAC |
| I | pKVM4-Check_fwd | CAATCGGATGCGATTATTG |
| J | pKVM4-Check-rev | TAGCTGTCGCTACTACTTTC |
| K | 00355_Check_fwd | GGATCTGGCACCTATGTAAG |
| L | 00355_Check_rev2 | ACGGCTCTTGTTGTAGTGG |
| M | 00355_Seq2 | CACCCGCAGGAACAAATG |
| N | 00355_Flank2_Seq | ATAAGTATCAAACAAGGCTATACTC |
| O | GroES_fwd | GTAGTGAATGTGATTAACAGCTGGTTTCTC AGCGGAAAAGAATGATGTAAGC |
| P | GroES_rev | ACCGGTAAACAGTTCTTCACCTTTGCTCAT TGAAATAACCTCCTCAATAGTATG |

**Table S3**: **Detection of qPCR signal of *gfp* gene in root samples at 33 dpi**

| Soil | Treatment | Number of replicates that showed qPCR signal |
| --- | --- | --- |
| Grass | Vegetative cells | **1** |
|  | Spores | **0** |
| ARD | Vegetative cells | **3** |
|  | Spores | **2** |

**Table legend:** Plantlets were inoculated with vegetative cells or spores of *P. megaterium* B1L5 and grown in grass or ARD soil. For each treatment and soil type, 6 replicates were analyzed. The number of replicates showing signal detection is indicated out of the total number of replicates (6).

**Table S4: Percentage of black root tips in apple plantlets grown in grass and ARD soil.**

| **Treatment** | **Grass soil** | **ARD soil** |
| --- | --- | --- |
| **Control** | 36 ± 22^aA^ | 75 ± 19**^a^**^B^ |
| **Vegetative cells** | 37 ± 11^a^*^A^* | 63 ± 16**^a^***^A^* |
| **Spores** | 25 ± 17^aA^ | 60 ± 4**^a^**^B^ |

**Table legend:** Plantlets were inoculated with either vegetative cells or spores of *P. megaterium* B1L5, or remained uninoculated as control. Values represent the mean ± standard deviation (SD) of the percentage of black root tips, calculated as (number of black tips / total number of root tips) × 100. Lowercase letters indicate statistical comparisons among the three treatments, "Control", "Vegetative cells", and "Spores", within the same soil type (**bold** letters for ARD soil and non-bold for grass soil). Uppercase letters denote comparisons of the same treatment between soils: “Control” in grass vs. ARD soil (no formatting), “Vegetative cells” (*italicized*), and “Spores” (underlined). Statistical significance was determined using Tukey’s HSD test at *p* < 0.05.

**Table S5: Plant growth parameters of apple plantlets grown in Grass or ARD soil.**

| Treatment | Soil | Shoot length (cm) | Total fresh mass (g) | Root fresh mass (g) |
| --- | --- | --- | --- | --- |
| Control | ARD | 5.07 ± 1.42**^a^**^A^ | 2.38 ± 0.77**^a^**^A^ | 0.96 ± 0.32**^a^**^A^ |
| Control | Grass | 3.95 ± 1.52^aA^ | 1.94 ± 0.37^aA^ | 0.81 ± 0.14^aA^ |
| Vegetative cells | ARD | 4.12 ± 1.13**^a^***^A^* | 2.16 ± 0.7**^a^***^A^* | 1.06 ± 0.37**^a^***^A^* |
| Vegetative cells | Grass | 6.03 ± 1.96^a^*^A^* | 2.84 ± 0.75^a^*^A^* | 1.38 ± 0.43^a^*^A^* |
| Spores | ARD | 3.6 ± 0.66**^a^**^A^ | 1.68 ± 0.57**^a^**^A^ | 0.7 ± 0.48**^a^**^A^ |
| Spores | Grass | 4.22 ± 0.87^aA^ | 2.28 ± 0.54^aA^ | 1.03 ± 0.44^aA^ |

**Table legend:** Plantlets were inoculated with either vegetative cells or spores of *P. megaterium* B1L5 or remained uninoculated as control. Values represent mean ± standard deviation (SD) for shoot length (cm), total fresh mass (g), and root fresh mass (g). Lowercase letters indicate statistical comparisons among the three treatments "Control", "Vegetative cells", and "Spores" within the same soil type (with **bold** letters representing ARD soil and non-bold for grass soil). Uppercase letters denote statistical comparisons for the same treatment across different soils: “Control” in grass soil vs. “Control” in ARD soil (no formatting), “Vegetative cells” in grass soil vs. “Vegetative cells” in ARD soil (*italicized*), and “Spores” in grass soil vs. “Spores” in ARD soil (underlined). Statistical significance was assessed using Tukey's HSD test at *p* < 0.05.

**Table S6: Significance of difference of composition of bacterial communities between different treatments was tested using the PERMANOVA test (*p* < 0.05).**

| Grass soil | | | | | |
| --- | --- | --- | --- | --- | --- |
|  | Df | SumOfSqs | R2 | F | Pr(>F) |
| Treatment | 2 | 0.50 | 0.19 | 5.28 | 0.001 *** |
| Time_Point | 1 | 0.47 | 0.17 | 9.89 | 0.001 *** |
| Treatment:Time_Point | 2 | 0.31 | 0.11 | 3.21 | 0.001 *** |
| Residual | 30 | 1.43 | 0.53 |  |  |
| Total | 35 | 2.70 | 1 |  |  |
| ARD soil | | | | | |
|  | Df | SumOfSqs | R2 | F | Pr(>F) |
| Treatment | 2 | 0.7 | 0.19 | 4.53 | 0.001 *** |
| Time_Point | 1 | 0.47 | 0.12 | 6.07 | 0.001 *** |
| Treatment:Time_Point | 2 | 0.30 | 0.08 | 1.95 | 0.004 ** |
| Residual | 30 | 2.32 | 0.61 |  |  |
| Total | 35 | 3.79 | 1 |  |  |

**Table S7: Significance of difference of composition of fungal communities between different treatments was tested using the PERMANOVA test (*p* < 0.05).**

| Grass soil | | | | | |
| --- | --- | --- | --- | --- | --- |
|  | Df | SumOfSqs | R2 | F | Pr(>F) |
| Treatment | 2 | 0.28 | 0.06 | 0.99 | 0.502 |
| Time_Point | 1 | 0.23 | 0.04 | 1.61 | 0.001 *** |
| Treatment:Time_Point | 2 | 0.31 | 0.06 | 1.09 | 0.2 |
| Residual | 30 | 4.23 | 0.84 |  |  |
| Total | 35 | 5.04 | 1 |  |  |
| ARD soil | | | | | |
|  | Df | SumOfSqs | R2 | F | Pr(>F) |
| Treatment | 2 | 0.27 | 0.047 | 0.82 | 0.865 |
| Time_Point | 1 | 0.27 | 0.048 | 1.67 | 0.003 ** |
| Treatment:Time_Point | 2 | 0.25 | 0.045 | 0.78 | 0.931 |
| Residual | 30 | 4.83 | 0.86 |  |  |
| Total | 35 | 5.62 | 1 |  |  |

**Supplementary figures**


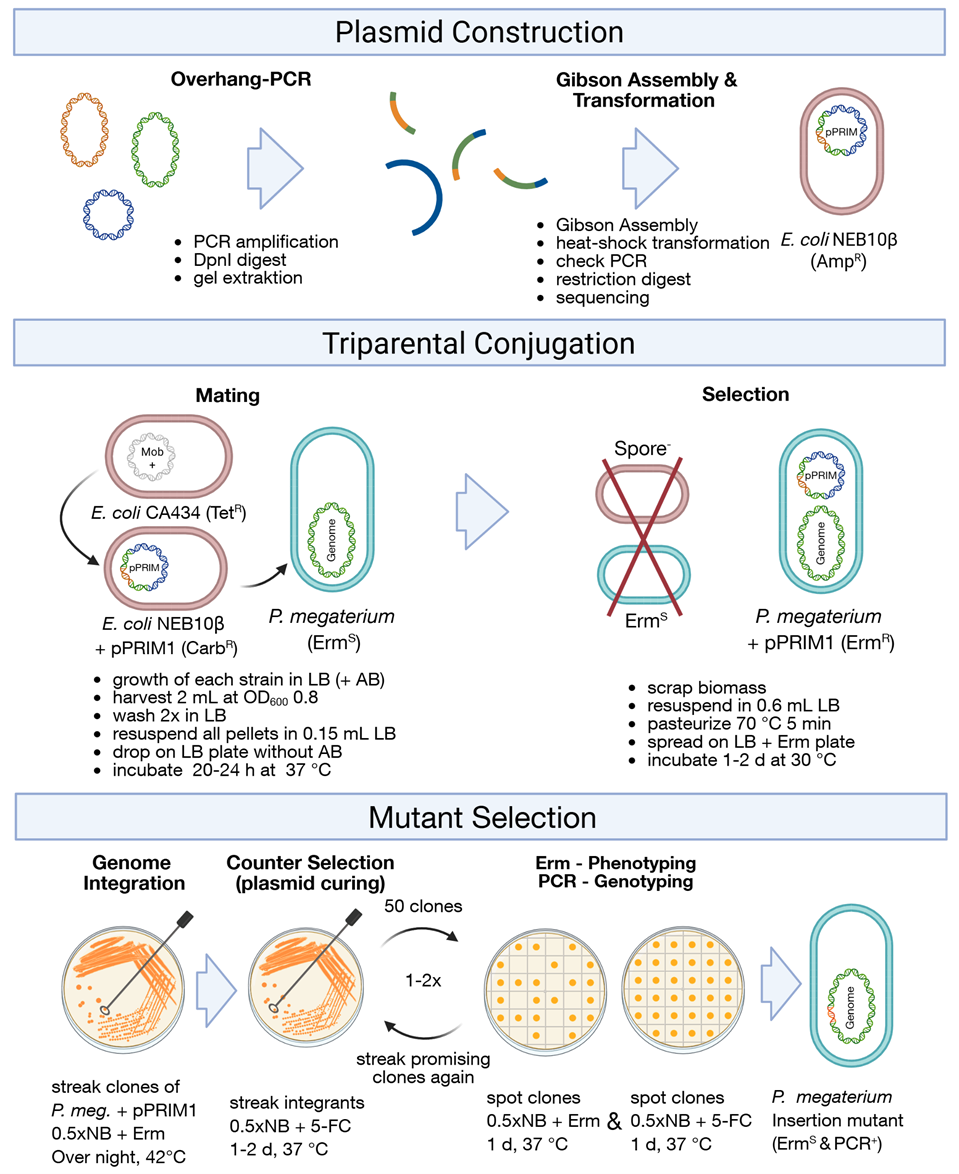


**Figure S1: Overview of construction of *P. megaterium* B1L5 in this study.** The process includes plasmid construction, conjugation, and final selection of the mutant. For a detailed description of the method, see the materials and methods section of the manuscript. The figure was created in BioRender. (<https://BioRender.com/>1z3za8k)


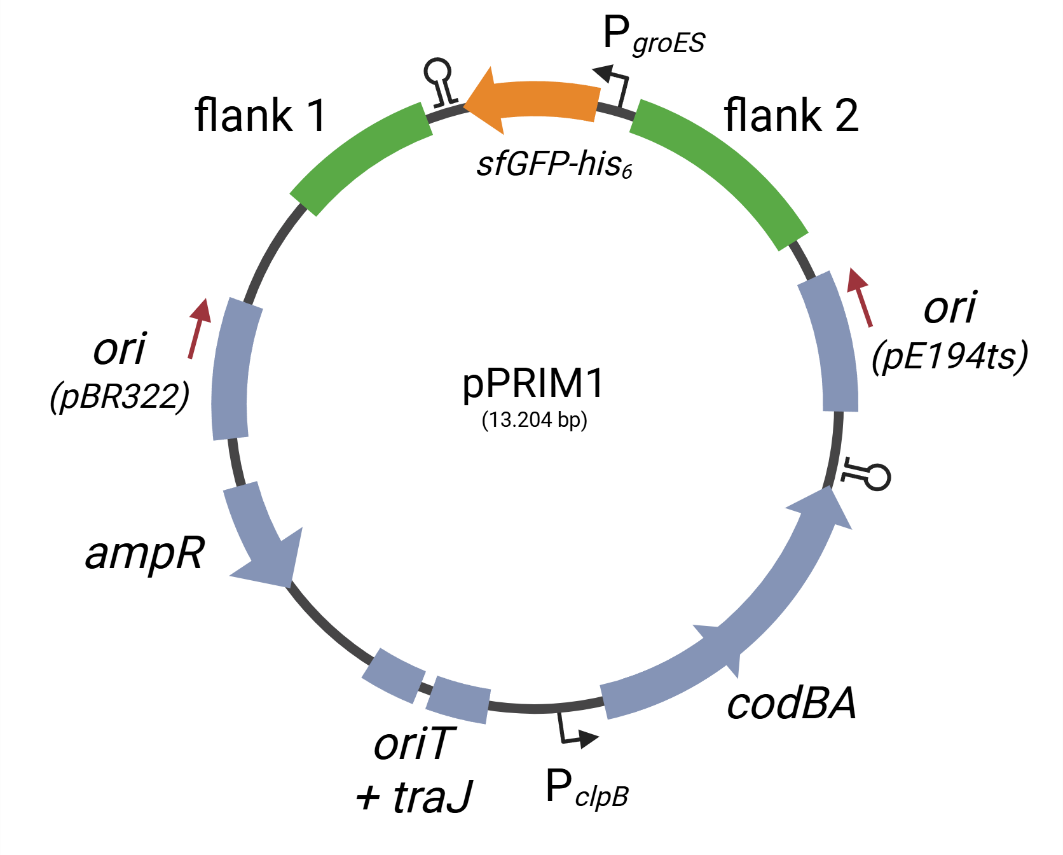


**Figure S2: Schematic representation of the vector pPRIM1.** The vector was used to integrate the synthetic DNA construct comprising *P_groES_* (*B. subtilis* 168) and *sfGFP*-His₆ with terminator (pBACOV-sfGFP) into the chromosome of *Priestia megaterium* B1. The plasmid was generated by inserting homologous flanking regions and synthetic expression construct into the vector pKVM4. The figure was created in BioRender. (https://BioRender.com/w73q762)

**Figure S3: Colonization of apple roots inoculated with *P. megaterium* B1L5 vegetative cells or spores and grown in grass soil.** CLSM images 6 **(A)** and 33 **(B)** dpi. White arrows points to bacterial cells. **(C)** Quantification of partial *gfp* gene copies per gram of root fresh mass, 6 dpi. The *p* value was determined using Wilcoxon test, and adjusted for multiple comparisons using the Benjamini-Hochberg (BH) method, using the rstatix v.0.7.2 R package.  For better visualization, a log10 transformation was applied to scale the y-axis.


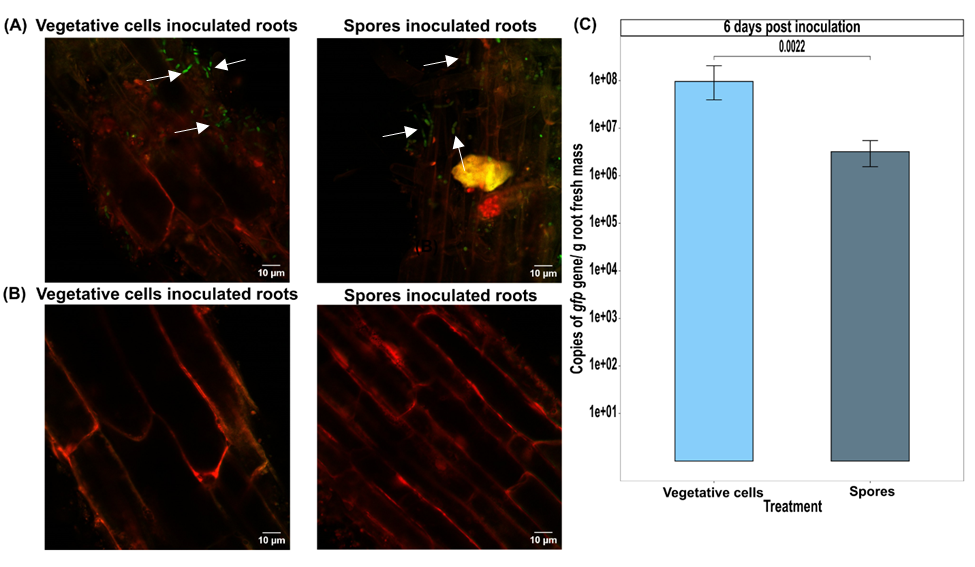

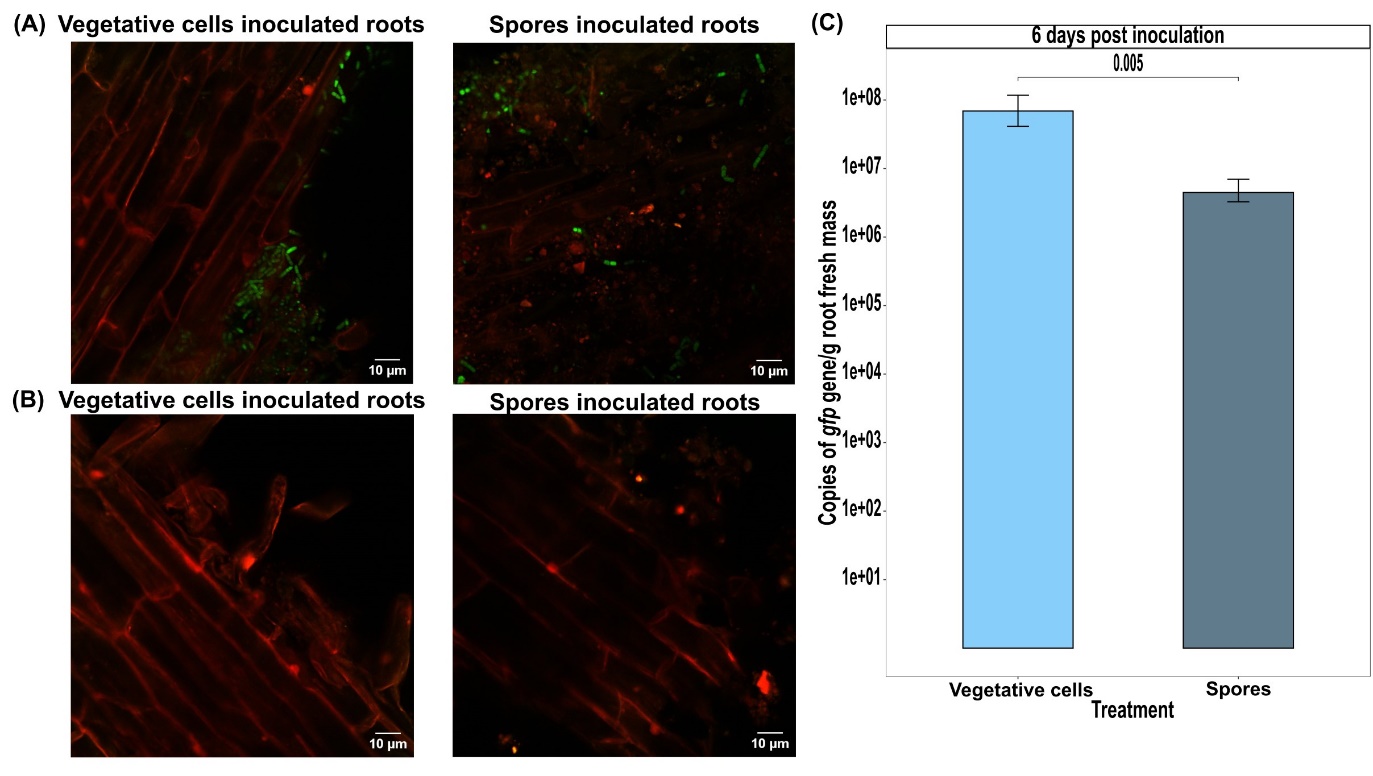


**Figure S4**: **Colonization of apple roots inoculated with *P. megaterium* B1L5 vegetative cells or spores and grown in ARD soil.** CLSM images 6 **(A)** and 33 **(B)** dpi. White arrows are pointing to bacterial cells. **(C)** Quantification of partial *gfp* gene copies per gram of root fresh mass, 6 dpi. The *p* value was determined using Wilcoxon test, and adjusted for multiple comparisons using the Benjamini-Hochberg (BH) method, using the rstatix v.0.7.2 R package.  For better visualization, a log10 transformation was applied to scale the y-axis.

**Figure S5: Richness and evenness of bacterial communities in grass or ARD soils. (A)** Richness indicated by Observed index. **(B)** Evenness indicated by Pielou index. Plantlets were inoculated with either vegetative cells or spores of *P. megaterium* B1L5 or remained uninoculated as control. Rhizosphere samples were collected 6 and 33 dpi. The *p* value was determined using Wilcoxon test, and adjusted for multiple comparisons using Benjamini-Hochberg (BH) method.


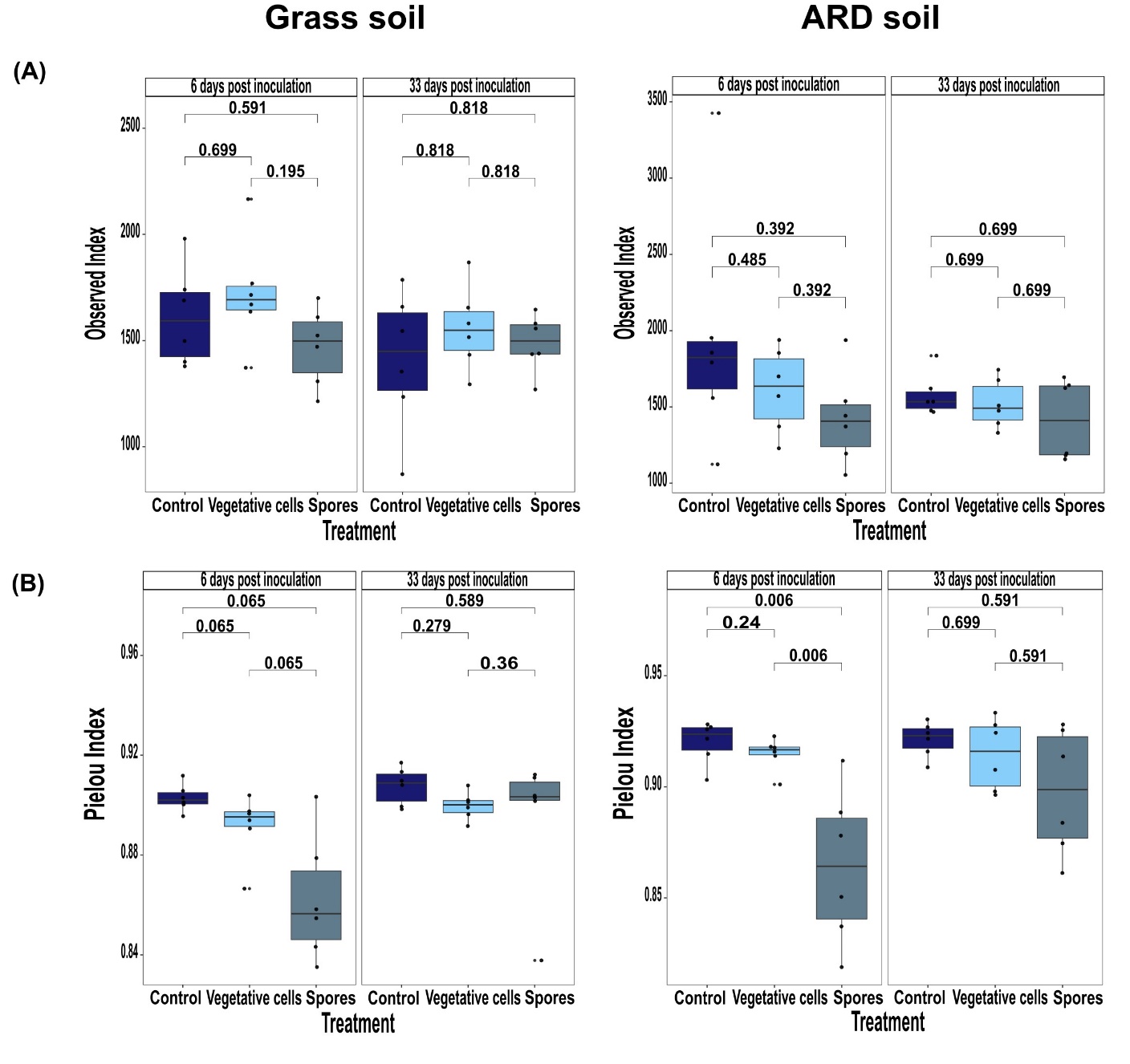

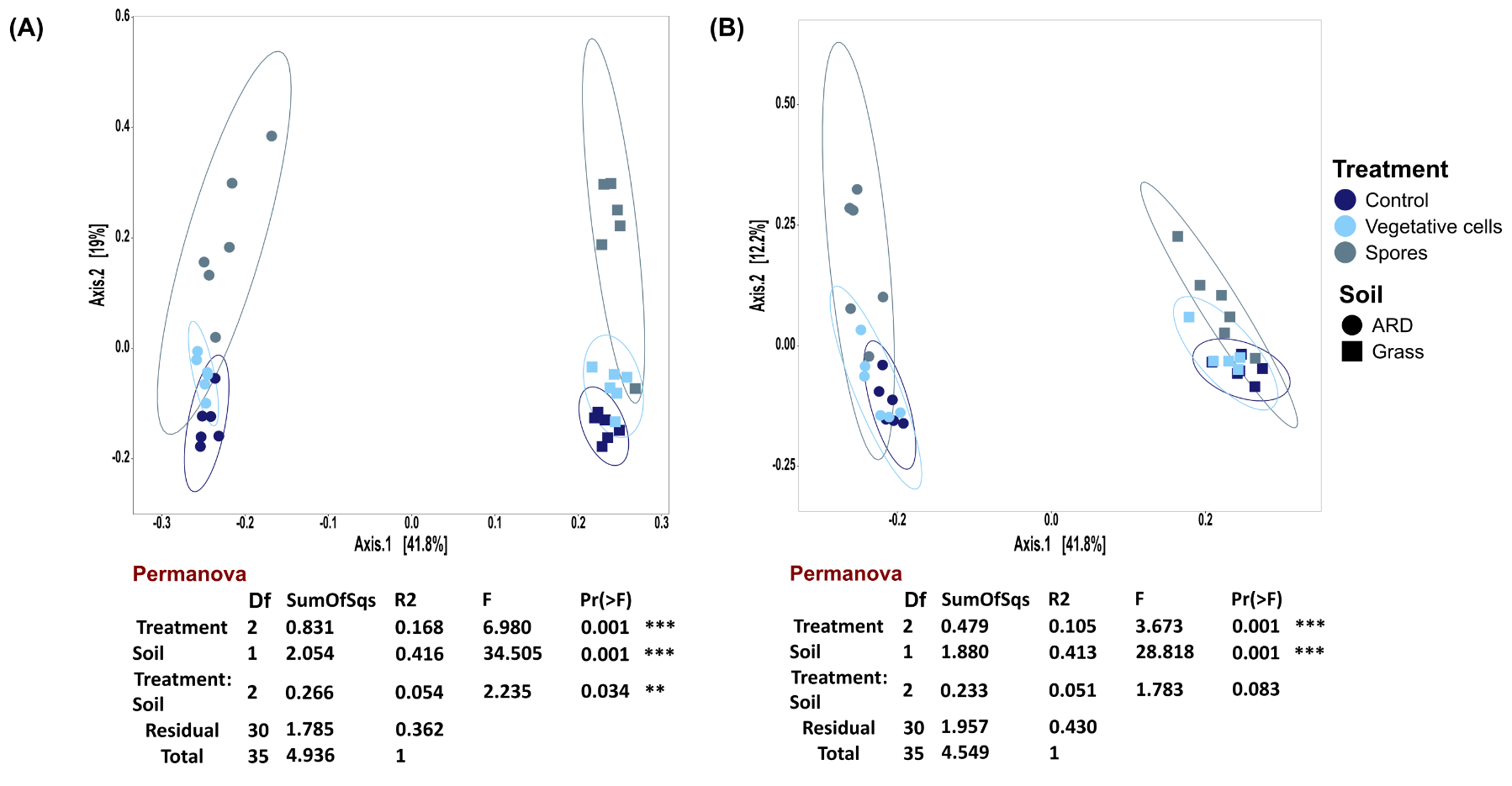


**Figure S6: Beta diversity of rhizosphere bacterial communities of apple plantlets grown in grass or ARD soil.** Plantlets were inoculated with either vegetative cells or spores of *P. megaterium* B1L5 or uninoculated controls and sampled at **(A)** 6 and **(B)** 33 dpi**.** Beta diversity is demonstrated by principal coordinates analysis (PCoA) based on Bray–Curtis distance. Significance of difference of composition of bacterial communities between different treatments and soils was tested using the PERMANOVA test (*p* < 0.05).


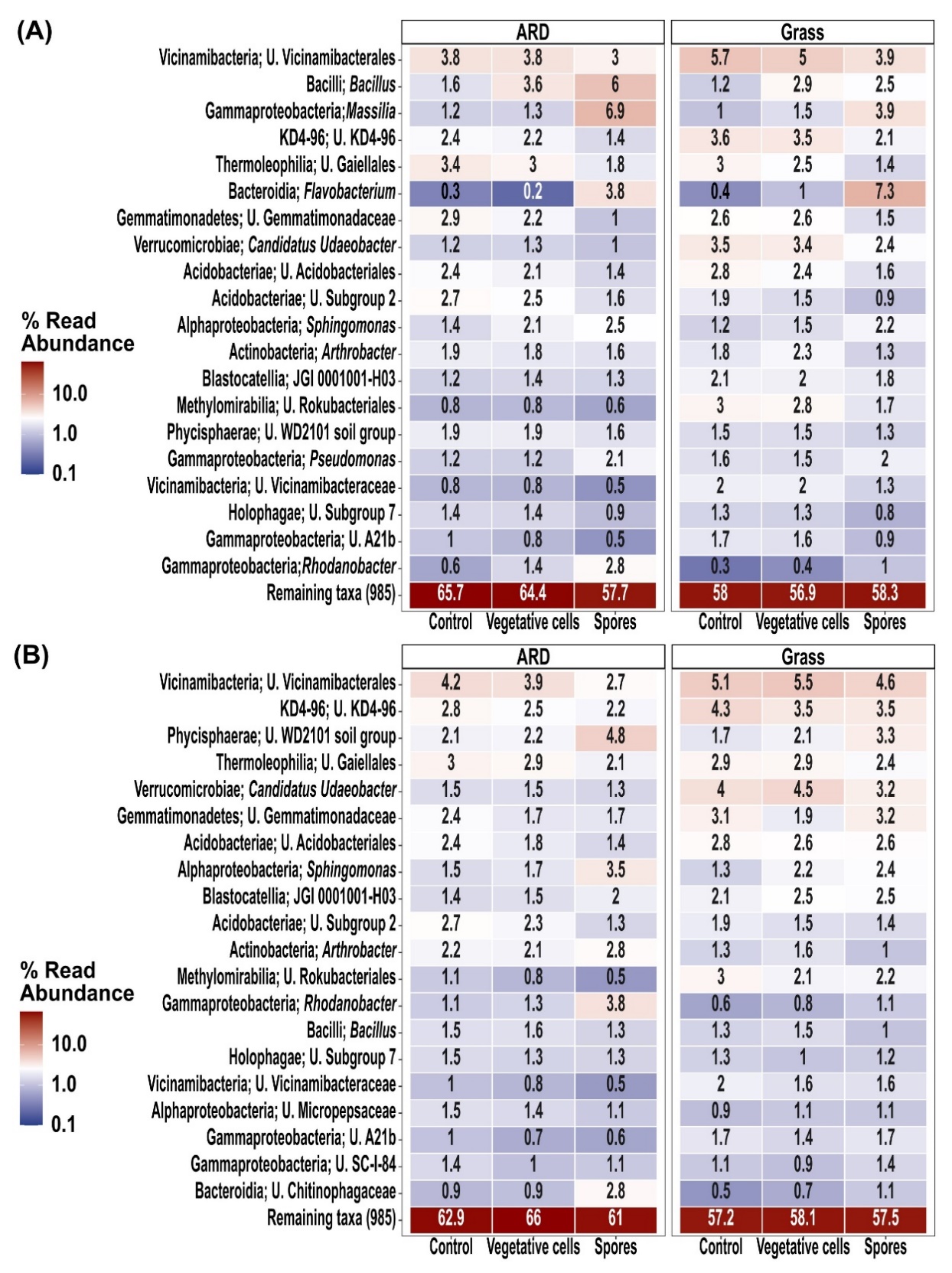


**Figure S7:** **Heatmap representing the relative abundance of top 20 abundant rhizosphere bacterial genera.** Plantlets were grown in ARD or grass soil and inoculated with either vegetative cells or spores of *P. megaterium* B1L5 or remained uninoculated as control, at **(A)** 6 and **(B)** 33 dpi. Values represent average relative abundance of 6 replicates of each treatment. U.’ denotes an unidentified genus within this higher taxonomic group. Names composed of numbers and letters represent taxa from groups lacking validly published scientific names, primarily sequenced through environmental studies.

**Figure S8:** **Differential abundance analysis of rhizosphere bacterial genera of uninoculated control apple plantlets grown in grass soil at 33 dpi compared to 6 dpi.** The analysis was performed using R package DESeq2 v.1.42.0. Log2-fold changes are shown on the x-axis. On the y-axis differentially abundant taxa with a *p* value < 0.05 are displayed.


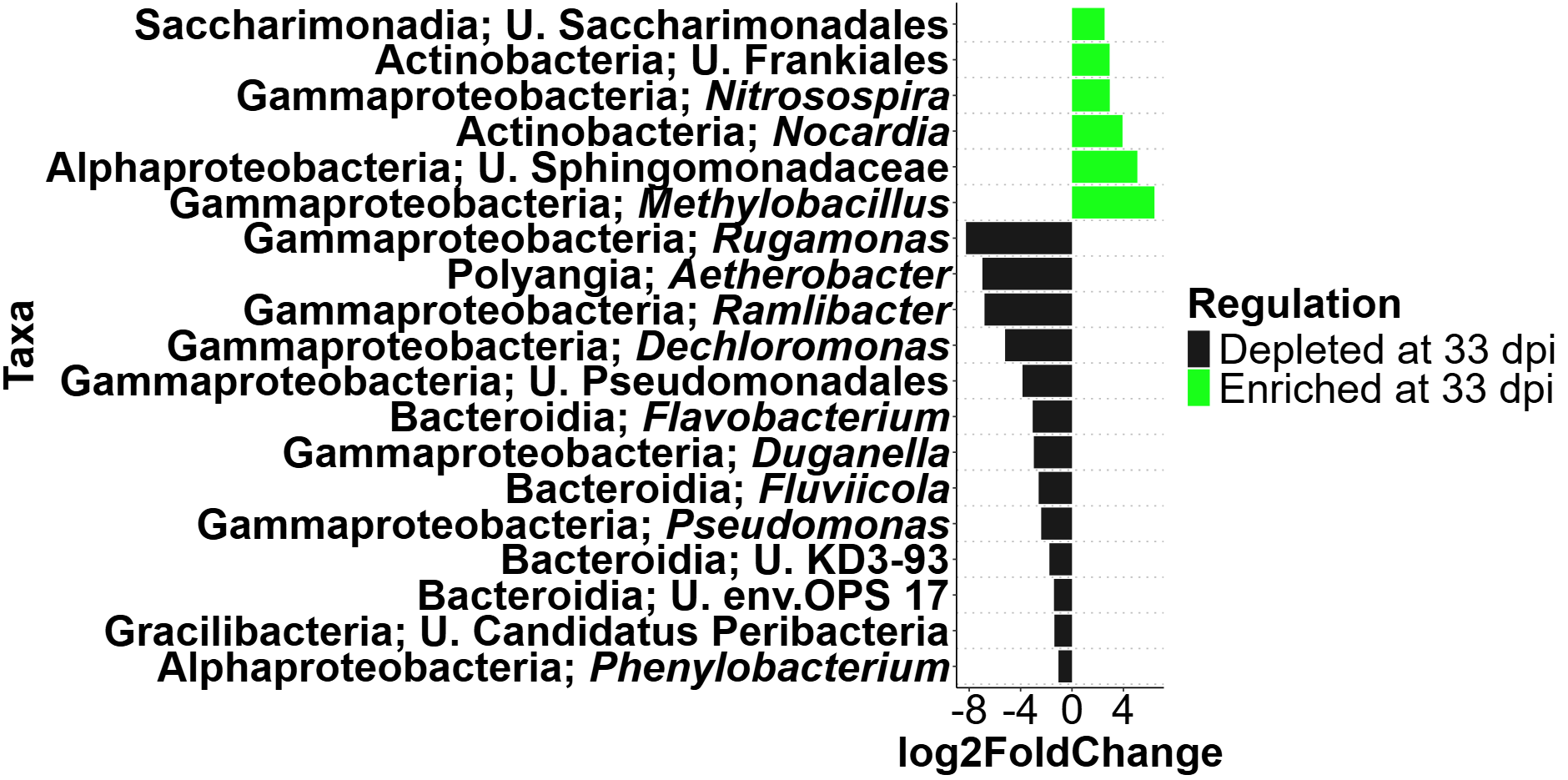


**Figure S9:** **Differential abundance analysis of rhizosphere bacterial genera of uninoculated control apple plantlets grown in ARD soil at 33 dpi compared to 6 dpi.** The analysis was performed using R package DESeq2 v.1.42.0. Log2-fold changes are shown on the x-axis. On the y-axis differentially abundant taxa with a *p* value < 0.05 are displayed.


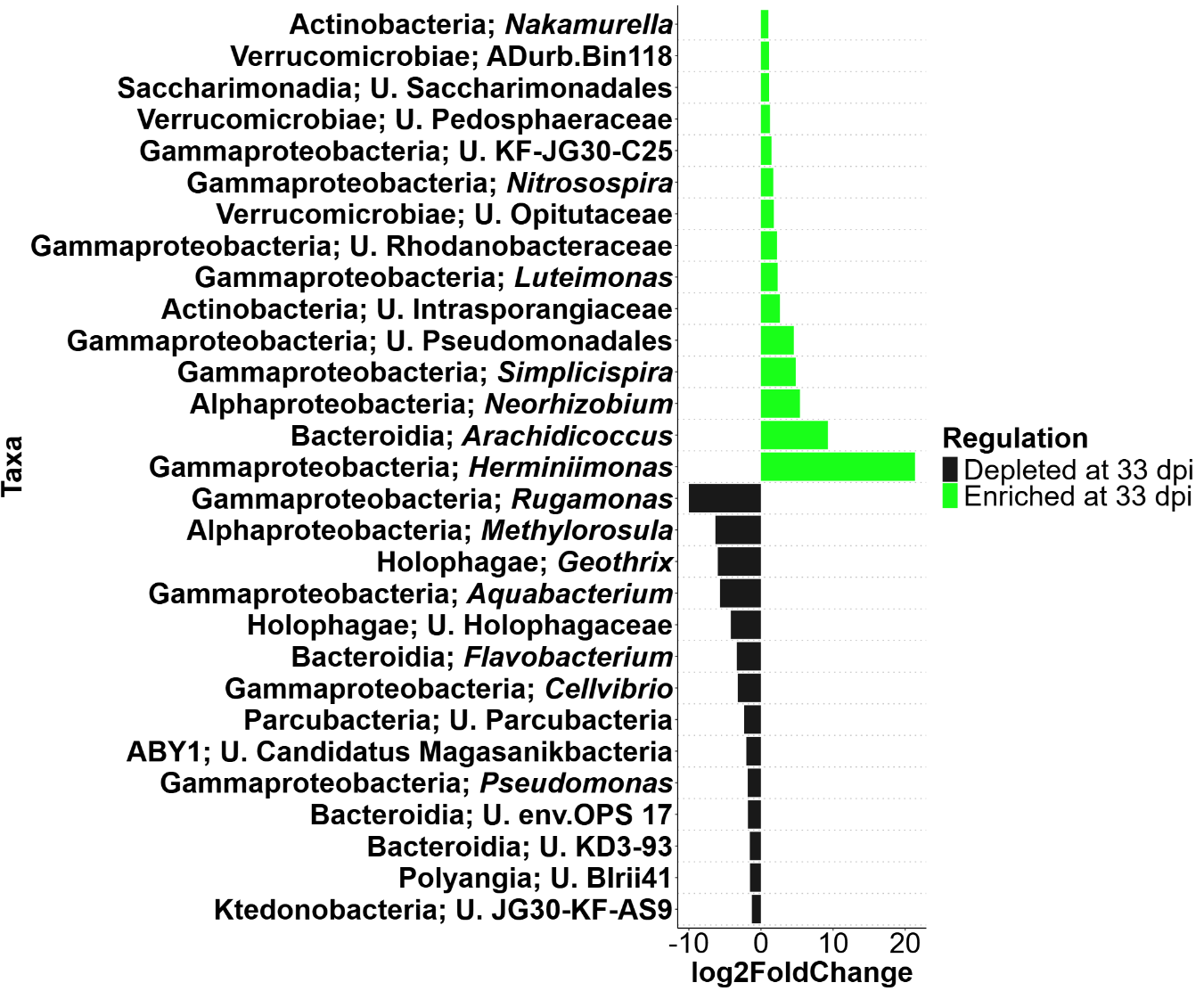

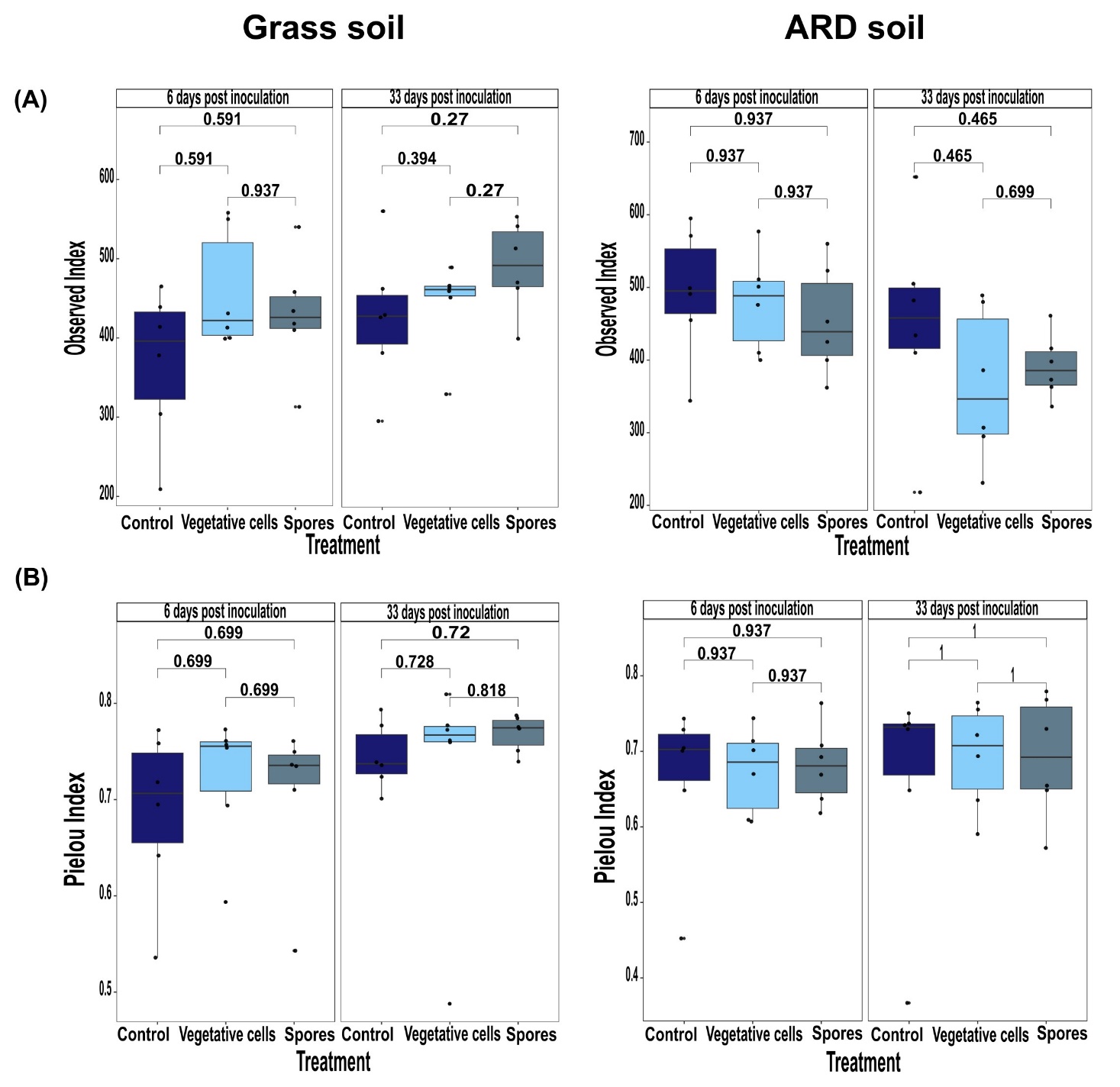


**Figure S10: Richness and evenness of fungal communities in grass or ARD soils. (A)** Richness indicated by observed index. **(B)** Evenness indicated by Pielou index. Plantlets were inoculated with either vegetative cells or spores of *P. megaterium* B1L5 or remained uninoculated as control. Rhizosphere samples were collected 6 and 33 dpi. The *p* value was determined using Wilcoxon test, and adjusted for multiple comparisons using Benjamini-Hochberg (BH) method.


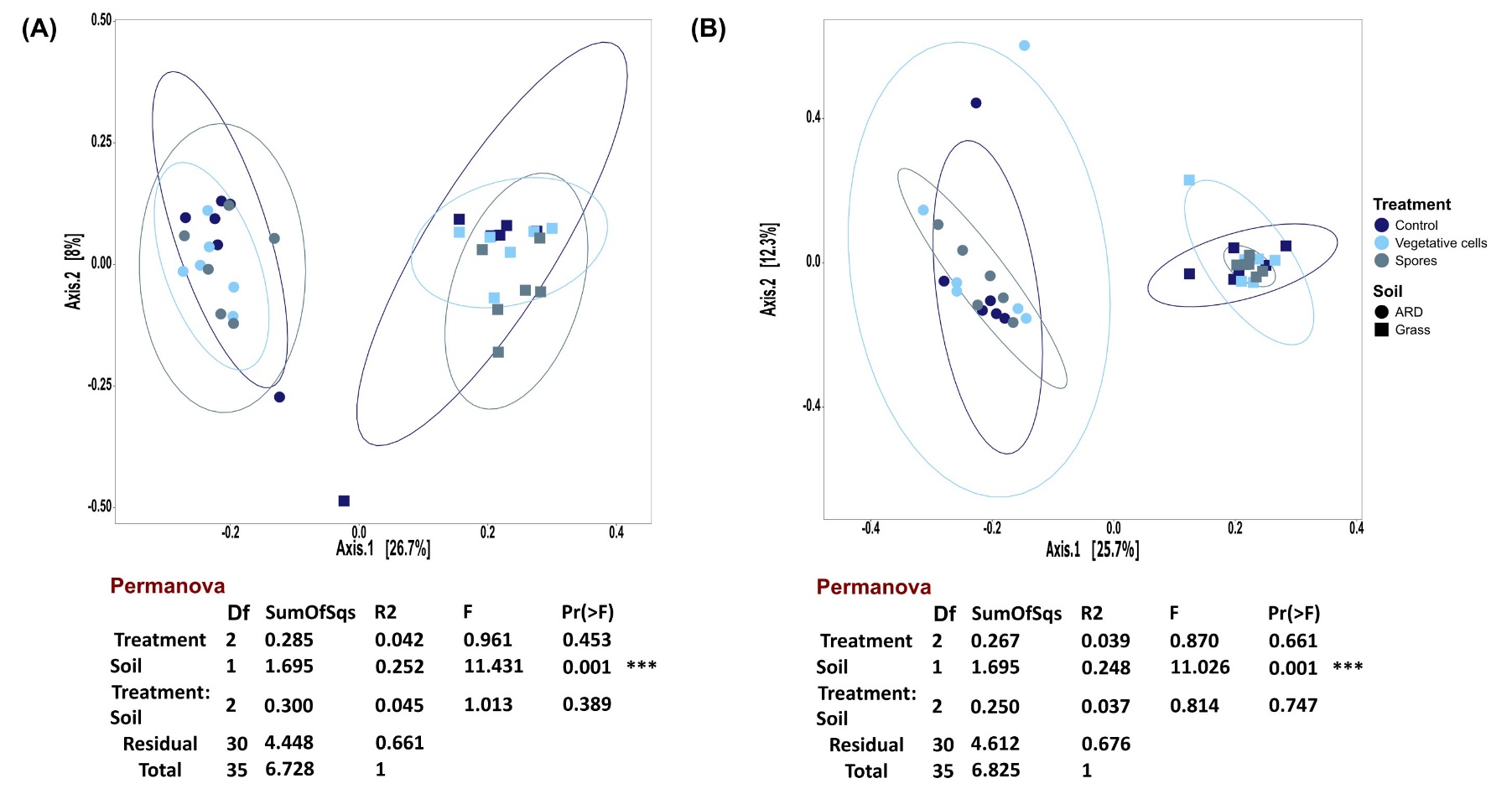


**Figure S11: Beta diversity of rhizosphere fungal communities of apple plantlets grown in grass or ARD soil.** Plantlets were inoculated with either vegetative cells or spores of *P. megaterium* B1L5 or remained uninoculated as control and sampled at **(A)** 6 and **(B)** 33 dpi**.** Beta diversity is demonstrated by principal coordinates analysis (PCoA) based on Bray–Curtis distance. Significance of difference of composition of fungal communities between different treatments and soils was tested using the PERMANOVA test (*p* < 0.05).


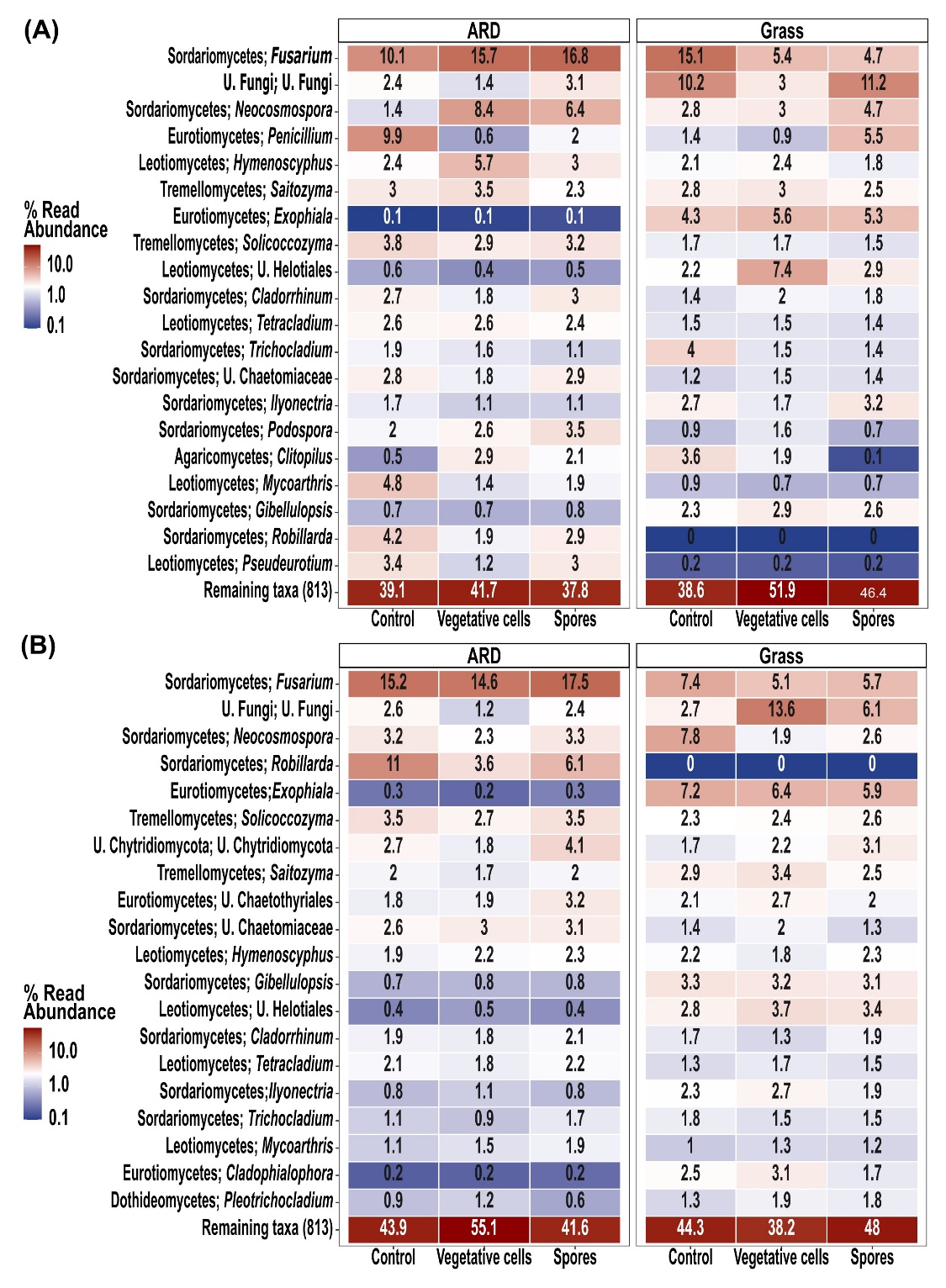


**Figure S12:** **Heatmap representing the relative abundance of top 20 abundant rhizosphere fungal genera.** Plantlets were grown in ARD or grass soil and inoculated with either vegetative cells or spores of *P. megaterium* B1L5 or remained uninoculated as control, at **(A)** 6 and **(B)** 33 dpi. Values represent average relative abundance of 6 replicates of each treatment. U.’ denotes an unidentified genus within this higher taxonomic group. Names composed of numbers and letters represent taxa from groups lacking validly published scientific names, primarily sequenced through environmental studies.

**Figure S13:** **Differential abundance analysis of rhizosphere fungal genera of uninoculated control apple plantlets grown in ARD soil at 33 dpi compared to 6 dpi.** The analysis was performed using R package DESeq2 v.1.42.0. Log2-fold changes are shown on the x-axis. On the y-axis differentially abundant taxa with a *p* value < 0.05 are displayed.


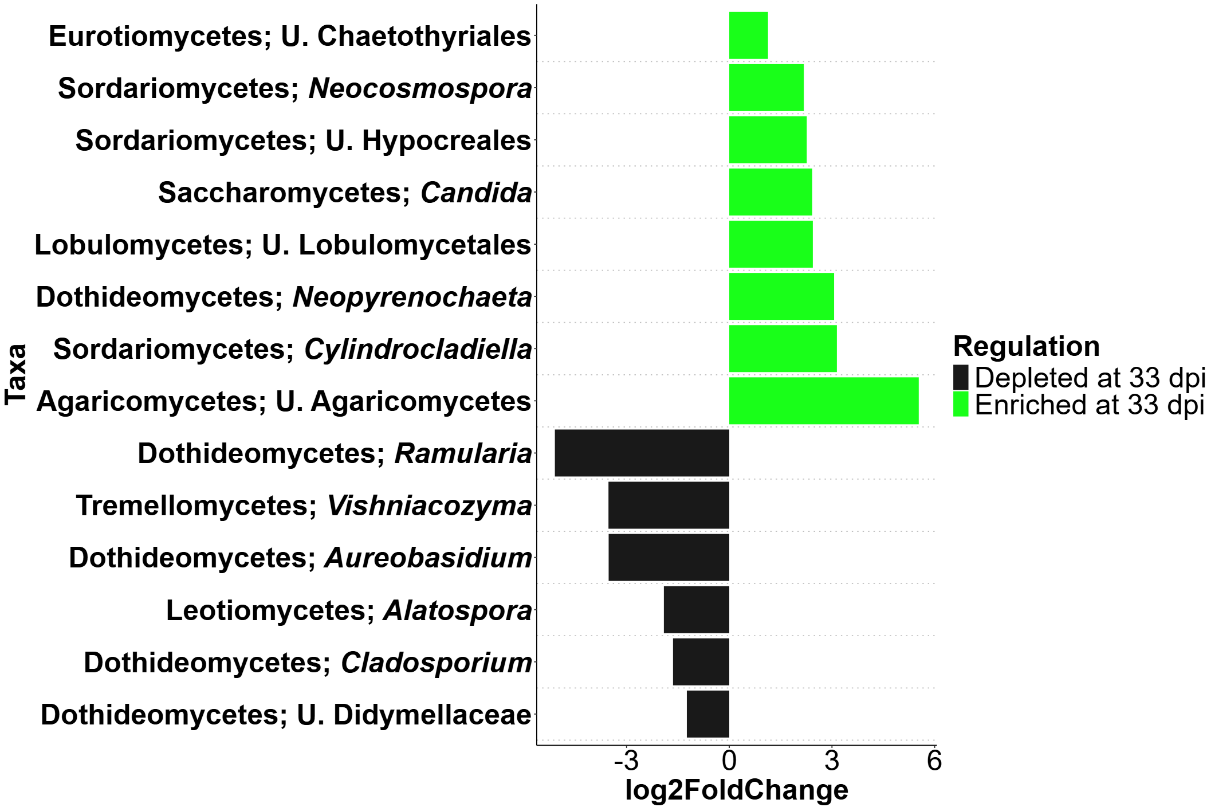

Supplement: Supplementary file 1 — Supplementary Material 1 [file 40793_2025_762_MOESM1_ESM.docx]
